# Genomic and transcriptomic analysis of the streptomycin-dependent *Mycobacterium tuberculosis* strain 18b

**DOI:** 10.1186/s12864-016-2528-2

**Published:** 2016-03-05

**Authors:** Andrej Benjak, Swapna Uplekar, Ming Zhang, Jérémie Piton, Stewart T. Cole, Claudia Sala

**Affiliations:** Global Health Institute, Ecole Polytechnique Fédérale de Lausanne, 1015 Lausanne, Switzerland; Current addresses: Department of Biology, Center for Genomics and Systems Biology, New York University, New York, NY USA; Current addresses: Department of Biochemistry, University of Lausanne, Quartier UNIL-Epalinges, Ch. des Boveresses 155, CH-1066 Epalinges, Switzerland

**Keywords:** *Mycobacterium tuberculosis*, Beijing family, Streptomycin-dependence, Translation initiation, 16S rRNA, IF3, Hypoxia, RNA-seq

## Abstract

**Background:**

The ability of *Mycobacterium tuberculosis* to establish a latent infection (LTBI) in humans confounds the treatment of tuberculosis. Consequently, there is a need to discover new therapeutic agents that can kill *M. tuberculosis* both during active disease and LTBI. The streptomycin-dependent strain of *M. tuberculosis*, 18b, provides a useful tool for this purpose since upon removal of streptomycin (STR) it enters a non-replicating state that mimics latency both *in vitro* and in animal models.

**Results:**

The 4.41 Mb genome sequence of *M. tuberculosis* 18b was determined and this revealed the strain to belong to clade 3 of the ancient ancestral lineage of the Beijing family. STR-dependence was attributable to insertion of a single cytosine in the 530 loop of the 16S rRNA and to a single amino acid insertion in the N-terminal domain of initiation factor 3. RNA-seq was used to understand the genetic programme activated upon STR-withdrawal and hence to gain insight into LTBI. This revealed reconfiguration of gene expression and metabolic pathways showing strong similarities between non-replicating 18b and *M. tuberculosis* residing within macrophages, and with the core stationary phase and microaerophilic responses.

**Conclusion:**

The findings of this investigation confirm the validity of 18b as a model for LTBI, and provide insight into both the evolution of tubercle bacilli and the functioning of the ribosome.

**Electronic supplementary material:**

The online version of this article (doi:10.1186/s12864-016-2528-2) contains supplementary material, which is available to authorized users.

## Background

Tuberculosis (TB) is a serious human disease caused by the airborne bacillus *Mycobacterium tuberculosis*. Responsible for over 1.5 million deaths and 9 million new cases of TB worldwide in 2013 [1], *M. tuberculosis* is arguably the most successful bacterial human pathogen ever. Over 2 billion individuals are estimated to be latently infected with *M. tuberculosis* [2], representing a huge reservoir for the emergence and spread of active TB that occurs in approximately 5–10 % of latently infected cases. The exact mechanisms and properties of latent TB infection (LTBI) are not fully elucidated. LTBI is represented by heterogeneous paucibacillary populations of *M. tuberculosis* with varying metabolic activities and replication rates, residing in tissues mostly without histological evidence of TB infection, and not necessarily restricted to pulmonary sites [3]. In essence, the pathogen can enter a dormant or latent state characterized by limited growth and metabolism, resulting in the absence of clinical symptoms in the host, and most importantly by increased phenotypic tolerance to the main drugs, thereby allowing indefinite persistence in the human body. This persistence is the main reason why the current treatment for new cases of pulmonary TB is very long, consisting of a six month therapy with four antibiotics (rifampicin, isoniazid, pyrazinamide, and ethambutol for the first 2 months, and only rifampicin and isoniazid for the last 4 months). In drug-resistant TB, the treatment duration is even longer and requires more expensive second-line drugs that are poorly tolerated and less effective than the front-line drugs.

To fight TB more efficiently, it is essential to shorten the treatment duration with new, more potent drugs that, ideally, are also active against LTBI. To facilitate the discovery of such drugs, *in vitro* models for LTBI can be used to screen chemical libraries. Current *in vitro* models such as nutrient starvation [4], nutrient depletion [5, 6], progressive hypoxia [7], nitric oxide treatment [8] and multiple stresses [9] mimic the dormant state of *M. tuberculosis* and are valuable for research purposes, but are impractical for high throughput applications.

The streptomycin (STR)-dependent *M. tuberculosis* strain 18b provides the basis of a simple and robust model that mimicks non-replicating bacteria. The strain was initially isolated as a STR-resistant mutant in Japan in 1955 [10] and then found to be STR-dependent. Strain 18b enters a viable but non-replicating state in the absence of STR and has been extensively validated as a simple drug discovery tool in our laboratory both *in vitro* and *in vivo* [11–14]. In addition, strain 18b has proved useful for vaccine studies and to investigate the basis of immunopathology in animal models [15–17]. Despite its success in biomedical research, little is known about 18b nor how well STR-starved 18b (SS18b) mimics LTBI compared to other dormancy models. In this work we determined and analysed the complete genome sequence of 18b and report the transcriptomic response to STR depletion.

## Results and discussion

### Whole genome sequencing and *de novo* assembly

The genome sequence was obtained by merging datasets generated using four different high-throughput sequencing platforms. Details of the coverage and the number of contigs obtained using each technology may be found in Table 1. The *de novo* assembly of the 454 dataset using Newbler v2.6 [18] produced a 4.4 Mb-long scaffold and two short scaffolds (5 and 3 kb). The 94 contigs obtained from the *de novo* assembly of the 454, Illumina and IonTorrent reads with MIRA v3.9.15 [19], were manually aligned onto the Newbler scaffolds in order to close gaps, since MIRA resolves repetitive areas more effectively. After this 22 gaps remained, all but one of which were closed using PacBio technology and HGAP2 software [20]. The remaining ~7 kb-long gap corresponds to the genes *rv3512* (*PE_PGRS56*), *rv3513c* (*fadD18*) and most of *rv3514* (*PE_PGRS57*). This area has the highest GC content in the H37Rv genome (79 %) and consists almost entirely of low-complexity and repetitive sequences. Read coverage and quality dropped dramatically for this genomic area for all sequencing technologies used, thus preventing accurate consensus calling, although from the available reads we could conclude that this area in 18b is similar to that in other *M. tuberculosis* strains.

**Table 1:** Sequencing and assembly of *M. tuberculosis* 18b genome

Sequencing			Assembly			
Sequencing platform	Average read length (nt)	Genome coverage	*Newbler* v2.6	*MIRA* v3.9.15	*HGAP2*	Manual finishing
454 PEs^a^	176	76x^b^	3 scaffolds	94 contigs	NA	1 contig
Illumina	35	48x	NA		NA	
IonTorrent	127	11x	NA		NA	
PacBio	2753^c^	79x^c^	NA	NA	4 contigs	

^a^3Kb and 8Kb paired-end libraries

^b^Excluding duplicate paired-end reads

^c^Filtered subreads

*NA* not applicable

The final assembly was obtained from the consensus of the three assemblies described above, resulting in a single contig containing 4.41 Mb. A total of 3930 protein coding-genes and 57 pseudogenes were predicted (see details below). The number of ribosomal RNA (3), tRNA (45) and other non-coding RNA genes (32) was the same as in the reference strain H37Rv.

### Phylogeny

**Fig. 1:**
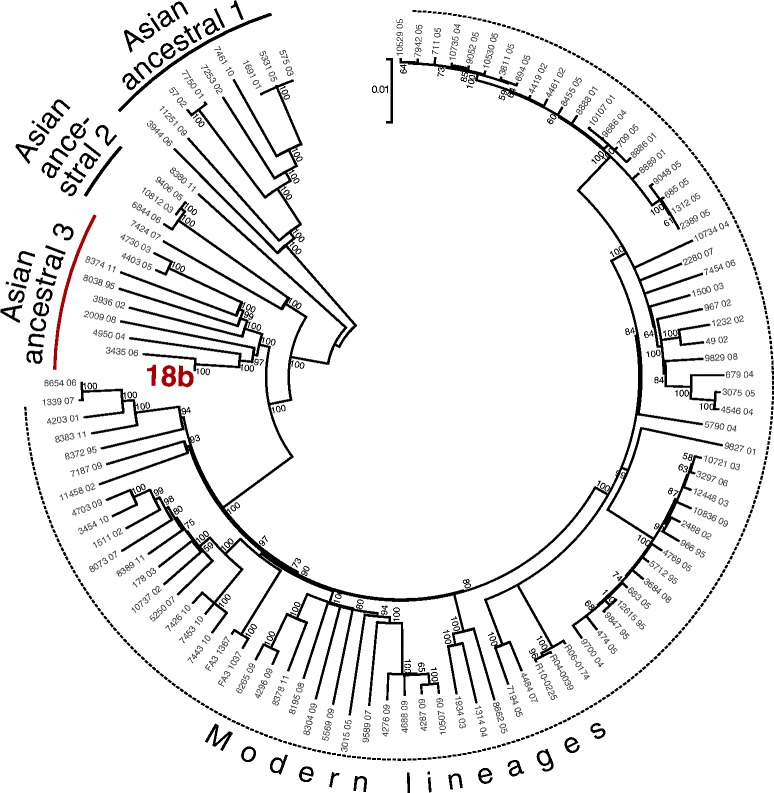
Maximum likelihood tree of 110 *M. tuberculosis* Beijing genomes [21] and the genome of 18b, based on 5992 SNP sites

To gain more insight into the origin of 18b we compared its genome sequence with the previously published SNP dataset of 110 *M. tuberculosis* strains from the Beijing lineage [21]. We obtained the same tree topology as Merker et al. [21], with 18b clustering within the “Asian ancestral 3” lineage (Fig. 1).

**Fig. 2:**
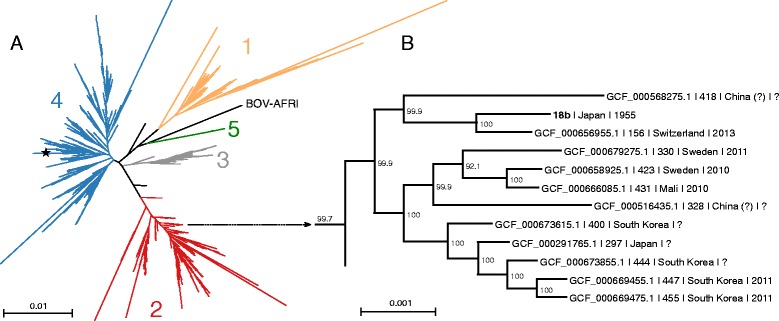
Phylogeny of *M. tuberculosis.* **a** SNP-based phylogenetic tree derived from the multiple alignments of 1794 *M. tuberculosis* genomes from the five main human lineages plus the *M. bovis*-West-Africa lineage (BOV-AFRI). Lineages were identified using the informative-SNP dataset from [22]. The star shows the position of the reference strain H37Rv. **b** A closer look at the 18b branch. For each sample, the accession number is given, followed by the total number of SNPs compared to the 18b genome, location of sample collection and year of collection

To further assess the relationship of 18b with other *M. tuberculosis* strains, we performed a phylogenetic analysis based on SNPs derived from whole genome alignments of 1793 *M. tuberculosis* strains deposited in GenBank. The phylogenetic tree (Fig. 2a) unambiguously distinguished all previously defined *M. tuberculosis* lineages [22, 23]. The strain that branched closest to 18b was TKK_04_0149 (NCBI assembly number GCF_000656955.1), isolated in 2013 in Switzerland. This strain differed by only 196 SNPs compared to 18b (Fig. 2b).

### Annotation and comparative genomics with strain H37Rv

The genome of 18b was annotated using multiple automated genome annotation engines, followed by manual curation (see Methods for details). We used orthoMCL [24] to infer the orthologs between H37Rv and 18b, and discarded singleton short hypothetical predictions (<100 codons) from the 18b annotation, as these generally result from overprediction by automated gene predictors. The remaining ortholog groups as well as genes unique to either H37Rv or 18b were manually checked. For the remaining longer predictions we retained only those supported by our RNA-seq data or by proteomics [25]. Finally we retained 21 novel gene predictions in 18b that were not present in the current H37Rv annotation (Additional file 1: Table S1).

Globally collinear with the H37Rv genome, the genome of 18b has 51 large indels (>100 bp) compared to H37Rv (Additional file 2: Table S2). Two deletions in the direct repeat (DR) region of 18b (>6 kb of sequence) produce the characteristic Beijing spoligotype, 000000000003771 [26]. Seventeen large indels were attributed to insertions or excisions of insertion sequences (IS), and 19 indels affected genes encoding proteins. The largest difference between the genomes of 18b and H37Rv is the absence of the PhiRv1 prophage from 18b, a 9245 bp area containing 15 genes (*rv1573-rv1587*) in H37Rv.

Fifty protein-coding genes present in H37Rv are missing from 18b (Additional file 3: Table S3), while 29 genes present in 18b have no ortholog in H37Rv (Additional file 4: Table S4). The majority of the missing genes correspond to phages, insertion sequences (IS) and hypothetical proteins. Exceptions are *rv1759c* (*wag22*) in H37Rv, a PE-PGRS family protein that is predicted to be secreted [27, 28] and *MT18B_4415* in 18b (molybdenum cofactor biosynthesis protein subunit MoaA; cyclic pyranopterin monophosphate synthase). Forty-four protein-coding genes were predicted as pseudogenes in 18b due to frameshifts or disruption by IS elements, while their orthologs in H37Rv are predicted to be functional (Additional file 5: Table S5). Consistent with 18b belonging to the Beijing family, *MT18B_2671* (*dosT*) was found to be frameshifted, as reported earlier by Fallow et al. [29].

Several genes were predicted to be longer in 18b than their orthologs in H37Rv. In most cases, an 18b gene overlapped two shorter open reading frames (ORFs) from H37Rv that are likely to be pseudogenes arising from a frameshift or a premature stop codon (Additional file 6: Table S6), but in some cases the difference was due to indels. For example, a curious case is the *kdpE* gene in 18b, which has a 35 bp deletion in its 3′-region leading to its fusion with the following ORF. Based on a BLAST search against all available *M. tuberculosis* sequences, the long version of *kdpE* was found to be exclusive to 18b.

The *MT18B_2492* gene, coding for isocitrate lyase (Icl), is intact in 18b, while it is frameshifted in H37Rv. Therefore, 18b possesses two functional isocitrate lyase genes (*icl1* and *icl2*), like the Erdman strain [30]. Another hallmark of the Beijing lineage is the presence of an intact *psk1-15* gene required for the production of the phenolic glycolipid PGL [31], in all other *M. tuberculosis* lineages this is frameshifted giving rise to *pks1* and *pks15* as exemplified by H37Rv. Other examples of “restored” ORFs in 18b include *PPE5*/*PPE6*, *mce2B*/*Rv0590A*, *mmpL13a*/*mmpL13b*, *celA2a*/*celA2b*, *pks3*/*pks4*, *rv3233c*/*tgs3*, *nat*/*rv3566A*, *fadD11.1*/*fadD11* etc. (Additional file 6: Table S6).

Another interesting case in *M. tuberculosis* 18b is that of *espK* (*MT18B_5142*/*rv3879c*), since the number of its characteristic tandem repeats, encoding GTPITPG motifs, is not only different between 18b and H37Rv, but also varies among other *M. tuberculosis* strains. EspK is an ESX-1 secretion-associated protein, and the ESX-1 secretion system is the major virulence determinant in *M. tuberculosis* and *M. marinum*, but the role of EspK in this system is not clear. Other genes that are significantly longer (>10 %) in 18b compared to H37Rv include *MT18B_3557*/*Rv2680*, *MT18B_4446*/*PPE54*, *MT18B_2455*/*Rv1888c*, *cobB*, *dxs2* and *lipV.* On the other hand there were 31 genes in 18b that were at least 10 % shorter than their orthologs in H37Rv, 15 of which coded for hypothetical proteins. In some cases, the difference in length was due to mutations, but in several others it was due to different predictions of translation start sites. We retained those start sites that were supported by our RNA-seq data. This was the case for 25 genes, of which 12 had a predicted function: *nat*, *fadD26*, *bioB*, *ispE*, *thiD*, *prfB*, *lipT*, *mrp*, *gcvT*, *ppiA*, *dop* and *sigG*.

Results from our comparative analysis point toward two major sources of gene variation among strains: gene prediction discrepancies, which can be considered as a technical problem, and genomic variations. Inconsistent gene predictions and annotations among closely related strains are a general problem, which could lead to errors in a naïve comparative approach. Accounting for this problem, we have identified several genes with different ORF lengths between 18b and H37Rv as a consequence of genomic variation. While the possible effect of such mutations could depend on many factors and so cannot be easily assessed *in silico*, it is plausible that some differences observed here might have a functional impact. Evidence of phenotypic diversity of *M. tuberculosis* strains, including important clinical traits such as virulence and pathogenesis is abundant [32–34], although linking specific mutations to an *M. tuberculosis* phenotype remains challenging especially for quantitative traits.

### IS elements

Except for IS*6110* and the PhiRv1 prophage, all the mobile elements identified in the genome of H37Rv [35] were also present in the genome of 18b, in the same genomic regions. IS*6110* is found exclusively in the *M. tuberculosis* complex where it is the most active IS element. It is therefore used as a diagnostic tool and for genotyping. The H37Rv genome contains 16 copies of IS*6110*, while 18b has 15 (Additional file 7: Table S7). Only five IS*6110* copies were found in the same genomic positions in both strains, but none of these loci was identical: one copy was truncated in 18b, three copies were inverted, and the synteny of the *plcD* region was altered due to recombination between flanking copies leading to only one copy of IS*6110* remaining in 18b. Another hallmark of the Beijing family is the presence of a copy of IS*6110* in the *dnaA-dnaN* locus [36].

### Streptomycin dependence and unique SNPs

**Fig. 3:**
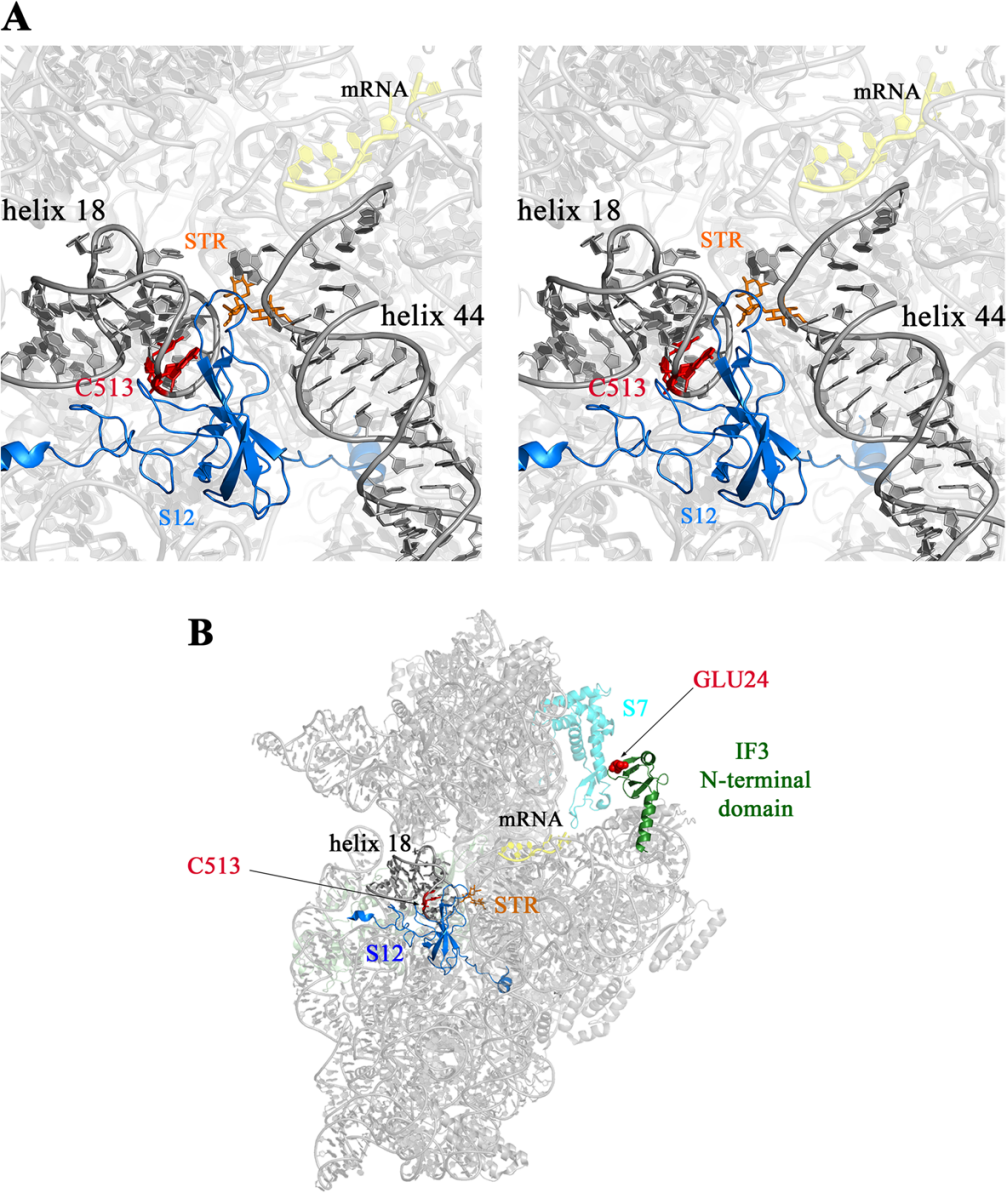
Structural model of the 30S subunit of the ribosome. **a** Stereoscopic view of the STR-binding site in the *T. thermophilus* 30S ribosomal subunit. STR, represented in orange, is in direct interaction with Helix 18 (530 loop) and Helix 44, both in dark grey, and the S12 protein in blue. In *M. tuberculosis* 18b, the cytosine inserted between G512 and C513 is shown in red. **b** Model of interaction between IF3 and the 30S ribosomal subunit. IF3N, represented in green with the insertion of Glu24 (in red), is in interaction with ribosomal protein S7 (in cyan)

The peculiar STR-dependent phenotype of 18b was initially attributed to a specific mutation in the *rrs* gene encoding the 16S rRNA [37]. The insertion of a cytosine (nucleotide position 512–513) in the 530 loop of 16S rRNA, a region known to be involved in STR susceptibility and resistance, seems to be exclusive to 18b since this mutation was not found in over 15,300 *M. tuberculosis* datasets from the Short Read Archive (SRA) at NCBI. Positions 512–513 in the 530 loop are in direct interaction with the ribosomal protein S12. Based on the structure of streptomycin bound to the 30S ribosomal subunit of *Thermus thermophilus* [38], we deduced that insertion of a cytosine at this position will affect the 530 loop conformation and could modify the binding of S12 and STR to the ribosome (Fig. 3a).

Curiously though, all our attempts to introduce this mutation into the H37Rv genome have failed, suggesting the presence of additional or compensatory mutations in 18b. Likewise, no streptomycin-susceptible revertants have ever been isolated, which is consistent with the hypothesis that STR-dependence may involve more than one mutation. Since STR inhibits translation we first examined all translation-related genes for the presence of SNP but only one was found in *fusA2*. This resulted in a missense mutation to elongation factor G (D170E) but this SNP is present in all strains from the Beijing family [22].

Assuming that such a second site mutation might be exclusive to 18b, we then extracted the 66 SNPs restricted to 18b from the comparison with 1793 *M. tuberculosis* genomes (Additional file 8: Table S8) and blasted each of the 20 bp regions encompassing them against 15,325 *M. tuberculosis* datasets from SRA. With the exception of one dataset (DRR014508, a clinical isolate from Japan), 64 out of 66 SNPs were very rare in *M. tuberculosis* datasets (Additional file 9: Dataset S1), and for the remaining two SNPs, both in *MT18B_2784* (*rv2113* ortholog), G2367949C and C2367951G, not a single read was found in any of the *M. tuberculosis* datasets. However, inspection of the genes associated with all 66 SNPs revealed no obvious link to streptomycin resistance.

Next, all genes harbouring small indels were examined leading to the finding that *infC*, encoding initiation factor 3 (IF3), had a single Glu codon inserted after codon 23. This insertion was not found in any of 1793 *M. tuberculosis* genome sequences available, nor in the 15,325 *M. tuberculosis* datasets from SRA, and affects a region of the protein that is well conserved among bacteria. IF3 is an essential protein that interacts with the 30S subunit of the ribosome, to which STR also binds. The inserted Glu residue in IF3 from strain 18b is located in the N-terminal domain, after turn one and at the start of beta-strand two in the 3D-structure of IF3 from *Geobacillus stearothermophilus* [39]. To date IF3 from mycobacteria has not been associated with STR-susceptibility or resistance. To go further, we modelled the interaction between the crystal structure of the 30S ribosomal subunit of *T. thermophilus* [38] and the N-terminal domain of IF3 (IF3N) by using the approximate orientation of IF3N derived from hydroxyl-radical cleavage data [40] (Fig. 3b). IF3 is localized near the E-site, far from the 530 loop, and does not seem to interact directly with streptomycin. However, IF3N is in contact with ribosomal protein S7, which has been implicated in conditional STR-dependence and makes translation hyper-accurate [41]. The inserted Glu24 seems to contact S7 and could thus affect its function leading to a STR dependent phenotype.

In prokaryotes, formation of the translation initiation complex requires binding of several components to the 30S ribosomal subunit namely, the mRNA, all three initiation factors and the f-Met initiator tRNA. STR typically binds to the 16S rRNA between the 530 loop (helix 18), helix 44, helix 27 and the ribosomal protein S12 and makes ribosomes error prone by affecting the proof-reading step. IF3, which has distinct N- and C-terminal domains, impacts binding of the other ligands and appears to play at least two roles [42]. IF3C prevents the association of the 30S and 50S ribosomal subunits whereas IF3N is implicated in verifying codon-anticodon complementarity [43] and docking studies have placed IF3N close to S7 and the peptidyl-tRNA binding site of the ribosome of *T. thermophilus* [44]. The structure of the *M. tuberculosis* ribosome is not yet available but should be similar to that of other prokaryotes (Fig. 3 [38]). Our finding of insertional mutations in both the decoding centre, the 530 loop, and in IF3N strongly suggests that these are compensatory changes required to allow translation to proceed in the presence of STR in strain 18b. The relationship between the two mutations is not direct but could have a strong impact on two important ribosomal proteins, S12 and S7.

### Transcriptional response of SS18b

In order to gain better knowledge of the biology of *M. tuberculosis* 18b and to characterize the transcriptional response to STR withdrawal we used RNA-seq to analyse gene expression in the exponential phase of growth with STR, and upon its removal, when *M. tuberculosis* 18b no longer grows and enters the non-replicating state (SS18b). Two time-points were chosen: 2 weeks after removal of STR, the standard time-point for evaluating drug activity [11–14], and 4 weeks. In addition, the antibiotic was added to the bacterial culture after 4 weeks without STR, leading to growth resumption, and RNA was then analysed. Sequencing reads were mapped to the *M. tuberculosis* 18b genome sequence. Overall, we used 2–4 biological replicates per condition. Additional file 10: Dataset S2 presents the results obtained. In the following paragraphs we will describe the main findings and compare the transcriptional response of SS18b to that of other models of non-replicating persistence (NRP).

Differential gene expression analysis was performed between the exponential growth phase and the non-replicating condition (2 and 4 weeks post STR-depletion). We detected 218 up-regulated and 193 down-regulated genes 2 weeks after STR withdrawal (at least 3-fold change at 1 % false discovery rate (FDR)) and 316 up-regulated and 418 down-regulated genes at 4 weeks (Additional file 10: Dataset S2). As previously shown [11], addition of STR to SS18b cultures after 4 weeks resulted in regrowth and almost completely restored the gene expression levels to those seen in exponential phase. Indeed, only 12 genes were found to be more expressed compared to the initial exponential phase (Additional file 10: Dataset S2). In general, the transcriptional response in SS18b after 4 weeks recapitulated that observed at week 2, although more pronounced differences were noted compared to the exponential growth phase (see below).

The two most up-regulated genes in SS18b, (*rv1057*, 40-fold-change, and *rv3289*, 38-fold-change) encode proteins of unknown function. *Rv1057* was induced in macrophage infection experiments [45, 46] but down-regulated during nutrient starvation [4] and not affected during low oxygen conditions [47]. On the other hand, transcription of *rv3289* increased in different dormancy models [4, 5, 46–49], but not in response to drugs [50].

Among the top up-regulated genes after 2 weeks of STR depletion were *sigE, sigB, sigL, dipZ* and *rv2877.* The sigma factor gene s*igB* is known to be up-regulated in stationary phase, under microaerophilic conditions [51], nutrient starvation [4, 5] and macrophage infection [45]. Genes belonging to the *cydABDC* operon, encoding cytochrome *bd*-type menaquinol oxidase, are among the most highly expressed genes in SS18b (11-14-fold change) and are also up-regulated during phosphate depletion [6] and microaerophilic condition [52]. Biogenesis of this oxidase requires DipZ.

Most heat-shock protein genes were induced in SS18b, notably *hspX* (12-fold), *htpX* (5-fold), *dnaK* (7-fold), *dnaJ2* (4-fold), and *hsp* (48-fold) whereas the most down-regulated genes were part of two operons: *rv0167*-*rv0178* (the Mce1 operon, 2-10-fold change) and *nuoA-N* (4-9-fold change). The latter, encoding the NADH-ubiquinone oxidoreductase (Complex I) involved in aerobic respiration, was repressed on nutrient starvation [4], upon phosphate depletion [6], during growth in macrophages and under low oxygen conditions [45, 52]. The exact function of the Mce1 operon is unknown, although it seems to be essential for survival in macrophages [53], and is down-regulated under low oxygen conditions and in *M. tuberculosis* inside macrophages [45, 52]. The second most repressed genes in SS18b were *recA* and *recX* (9-fold change). A Himar*1*-insertion mutant of *recA* is known to produce slow growing colonies [54] and the *recAX* operon to be silenced upon phosphate depletion [6] or hypoxia [47].

Transcription of the molybdopterin biosynthesis locus, *moa1,* was down-regulated in SS18b (3-8-fold less abundant compared to exponential phase), while the *moa2* locus was not significantly affected. The *moa1* cluster was down-regulated in low oxygen conditions [47], but increased transcription was reported during phosphate depletion [6] and in murine macrophages [52]. The *trp* operon (*rv1609-rv1614*) is down-regulated in SS18b and was also reported as being repressed under different low oxygen conditions [52].

Some non-coding RNAs (ncRNAs) were described as differentially expressed during stationary phase in previous studies. Notably, *mcr11* and MTS2823 were up-regulated in stationary phase [55, 56]. In our study, *mcr11* (MT18B_7019) was up-regulated 8-fold while MTS2823, the most abundant ncRNA in *M. tuberculosis* H37Rv, was up-regulated 4-fold. Other ncRNA genes were significantly induced after STR removal (>3-fold): *mcr19* (*MT18B_7000*), *ncrMT3949* (*MT18B_7009*), *mpr6* (*MT18B_7002*), *mcr10* (*MT18B_7023*), and *AS1890* (*MTB000056*). On the contrary, *ncrMT1234* (*MT18B_7008*) and *MTS2975* (*MT18B_7029*) were repressed when bacteria did not grow and an antisense RNA from the *ino1* gene was not found under any growth conditions, unlike in strain H37Rv [57, 58].

After 4 weeks of STR depletion more genes were deregulated, including most of those found to be differentially expressed at week two but with some exceptions (Additional file 10: Dataset S2). The most notable were *rv2016*, *glbN*, *ahpC, higA* and *higB*, which were >7-fold up-regulated after 2 weeks of STR depletion but virtually restored to the control levels after 4 weeks. Such marked differences were not observed for the down-regulated genes.

Some genes were even more significantly differentially expressed after 4 weeks of STR depletion. For example, *rv1066* (16-fold up-regulation after 2 weeks, and 109-fold up-regulation after 4 weeks), *erm(37), dut*, *MTS2823* and *rv2696* (3-4-fold up-regulation after 2 weeks, and 20-26-fold up-regulation after 4 weeks), *rv1514* (4-fold down-regulation after 2 weeks, and 19-fold down-regulation after 4 weeks).

### Comparison with other models of NRP

We compared our data genome-wide with those obtained with other models of NRP: the nutrient starvation model [4], the oxygen depletion condition [59], and various stresses mimicking the non-replicating or persistent state [6, 8, 9, 47–49, 60]. The comparisons are summarized as weighted Venn diagrams in Additional file 11: Figure S1. Despite differences in the experimental conditions and statistical methods used, we observed a certain degree of overlap and consistency between the various models, especially for the direction of gene regulation (i.e. up- or down-regulation). For example, most of the genes defined as “dormancy regulon” by Voskuil et al. [8] or additional “stationary-phase-induced” genes [47] are also up-regulated in SS18b, albeit most of them having an FDR over 5 %. Better overlap with the SS18b response was observed for the results from microaerophilic rather than prolonged anaerobic conditions [48, 49, 59], consistent with growth of 18b in the presence of air. Interestingly, the transcriptional response of *M. tuberculosis* growing in macrophages was in great part consistent with that of STR depletion in 18b. Importantly, while the other studies employed microarrays, ours represents the first to use RNA-seq, thus providing higher resolution and detection of otherwise missed small transcripts.

**Fig. 4:**
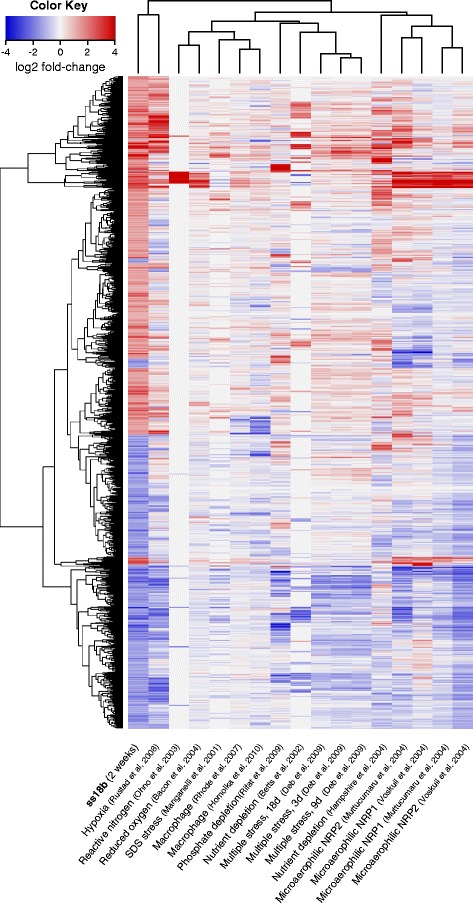
Hierarchical clustering of differentially expressed genes in SS18b and the results from previous works. Up-regulated genes are in red, down-regulated in blue. Red and yellow arrow heads denote “dormancy regulon” genes as defined in [8] and [47] respectively. Only genes that were at least two-fold differentially expressed (regardless of the FDR) in 18b were considered

Some genes are consistently regulated in various dormancy models [8, 47, 52]. To see if a similar pattern would emerge using our data we performed a hierarchical gene clustering analysis of the SS18b differentially expressed genes (2-fold-change cut-off) with those of other NRP models (Fig. 4). The strongest clustering occurred for the “dormancy regulon” genes [8]. The datasets from microaerophilic NRP conditions clustered together as expected. Expression signatures from the multiple stress method [9] clustered together with those of phosphate [6] and nutrient depletion [4], which can be attributed to the fact that nutrient starvation had the strongest effect in that multi-stress study [9]. Interestingly, the results from one hypoxia study [59] clustered closest to SS18b, but those of other hypoxia models were scattered (Fig. 4). Additional datasets can be found in Additional file 12: Figure S2B, Additional file 13: Figure S3B and Additional file 14: Figure S4.

### *M. tuberculosis* 18b-specific features

Comparing our results with those derived from other conditions enabled us to identify genes that are uniquely expressed in the SS18b model. For example, *rv1066, rocA*, *pepE*, *rv3143*, *PE_PGRS49*, *rv0106* and *rv1084* were at least 4-fold up-regulated, but either unaffected or down-regulated in other dormancy models. *Rv3143* was described as upregulated in a *∆dosR* mutant [61] and in MDR strains [62]. On the other hand, *rv0106* was induced by stationary phase [47] but not in non-replicating persistence models. Similarly, *rv2719c*, *rv3201c*, *rv3611*, and *rv3108* were at least 4-fold down-regulated in SS18b, but either unaffected or up-regulated in other studies. Both *rv2719c* and *rv3201c* were found to be induced by DNA damage [63], whereas *rv3611* encodes for an essential putative antigen. Finally, *rv3108* is located upstream of the *moa* operon, which is repressed in SS18b.

## Conclusion

The genome analysis presented in this study shows strain 18b to belong to the Beijing family of *M. tuberculosis* and, in particular, to clade three of the ancient ancestral lineage. In addition to the genetic markers characteristic of the Beijing family, such as an intact *psk1-15* gene and polymorphisms in the *fusA2* and *dosT* genes, strain 18b has an unusual frameshift mutation in its *kdpE* gene encoding the transcriptional regulatory protein KdpE. Loss or altered function of this part of the two-component system, KdpDE, may result in an altered response to potassium limitation due to aberrant regulation of the linked *kdpFABC* operon encoding a potassium transport system. The DNA binding domain of KdpE is situated at the C-terminal end of the protein; however, in 18b, this domain is followed by an additional 220 amino acid residues. Functional investigation into potassium uptake is ongoing.

A major finding of this investigation was the discovery of a second mutation that may contribute to the unique STR-dependence. In addition to the insertion of a cytosine in the 530 loop region of the 16S rRNA [37], we report a mutation in *infC*, coding for initiation factor IF3. Our modelling studies provided a potential explanation to the role played by this mutation in STR-dependence. Experimental support is now required for this interpretation, which provides new insight into ribosome function.

From the comparative transcriptomic study of SS18b and other NRP models of *M. tuberculosis* we were able to draw the following conclusions. First, upon STR-removal gene expression is altered thereby allowing cell metabolism to adapt to the non-replicating state. This is reflected in a shift from aerobic growth as evidenced by high NADH-oxidase levels to microaerophilic conditions with induction of cytochrome *bd*-type menaquinol oxidase and the components of the dormancy regulon. Overall, although there are some particularities, the gene expression profiles in SS18b are reasonably consistent with those observed in other NRP models and with the transcriptional profile of *M. tuberculosis* growing intra-cellularly. This once again underlines the utility of strain 18b as a model for understanding NRP on the one hand and for finding drugs active against LTBI on the other.

## Methods

### Bacterial strains and culture conditions

*M. tuberculosis* 18b was grown at 37 °C with shaking in 7H9 broth (Difco) supplemented with 10 % albumin-dextrose-catalase (ADC) enrichment, 0.2 % glycerol, 0.05 % Tween 80, 50 μg/ml STR or on solid Middlebrook 7H10 medium (Difco) supplemented with 0.5 % glycerol, 10 % oleic acid-albumin-dextrose-catalase (OADC), 50 μg/ml STR. Non-replicating, STR-starved 18b (SS18b) was generated as follows. 18b was grown to mid-logarithmic phase in STR-containing medium and washed three times in phosphate-buffered saline containing 0.05 % Tween 80 (PBST). The final bacterial pellets were resuspended in medium without STR and maintained at an optical density at 600 nm (OD_600_) between 0.2 and 0.5 for 2 weeks (with the addition of fresh medium if necessary), by which time they had stopped replicating.

### Genomic DNA preparation

*M. tuberculosis* 18b was grown in 7H9 complete medium to OD_600_ 0.8, then 10 ml of culture were centrifuged, cells were resuspended in 250 μl SET (25 % sucrose, 50 mM EDTA, 50 mM Tris HCl pH8) and 50 μl of 20 μg/ml lysozyme added. After overnight incubation at 37 °C, the suspension was treated first for 30 min at 37 °C with 5 μl of 10 mg/ml RNAse A and then for 2 h at 55 °C with 250 μl Proteinase K solution (400 mg/ml Proteinase K, 100 mM Tris HCl pH8, 0.5 % SDS). DNA was extracted once with phenol-chloroform-isoamyl alcohol (25:24:1), once with chloroform-isoamyl alcohol (24:1), precipitated in ethanol, air-dried and resuspended in TE buffer. The amount, integrity and purity of DNA were checked using Nanodrop and Qubit instruments (Life Technologies), and electrophoresis on an agarose gel (0.6 % w/v).

### RNA preparation

Replicating (i.e. with STR) or STR-starved *M. tuberculosis* 18b cultures (40 ml) were pelleted and cells flash frozen in liquid nitrogen and stored at −80 °C until use. Bacteria were re-suspended in 1 ml Trizol (Invitrogen) and added to a 2-ml screw-cap tube containing 0.5 ml zirconia beads (BioSpec Products). Cells were disrupted by bead-beating twice for 1 min with a 2-min interval on ice. The cell suspension was then transferred to a new tube, where chloroform-isoamylalcohol (24:1) extraction was performed. RNA was precipitated by adding 1/10 volume of sodium acetate (2 M, pH 5.2) and 0.7 volume of isopropanol, washed with 70 % ethanol, air-dried and resuspended in DEPC-treated water. DNase treatment was carried out twice using RQ1 RNase-free DNase (Promega), following the manufacturer’s recommendations, and the reactions were subsequently cleaned up by phenol-chloroform extraction and ethanol precipitation. RNA was stored at −80 °C in DEPC-treated water. Amount and purity of RNA were determined spectrophotometrically and by Qubit analysis (Life Technologies), integrity of RNA was assessed on 1 % agarose gel and by Fragment Analyzer (Advanced Analytical).

### Library preparation for Illumina high-throughput sequencing

Sequencing libraries were prepared using the TruSeq DNA Sample Prep Kit (Illumina) according to the protocol supplied with the reagents and using 1 μg of genomic DNA purified as described above. The resulting genomic DNA fragment library was loaded onto one channel of a single read v4 flowcell and sequenced on the Illumina Genome Analyzer IIx using the 36 Cycle TruSeq SBS Kit v5. Data were processed using the Illumina Pipeline Software package v1.7.

### Library preparation for Pacific Biosciences high-throughput sequencing

High molecular weight DNA from *M. tuberculosis* was sheared in a Covaris g-TUBE (Covaris, Woburn, MA, USA) to obtain 20 kb fragments. After shearing the DNA size distribution was checked on a Fragment Analyzer (Advanced Analytical Technologies, Ames, IA, USA). 5 μg of the sheared DNA was used to prepare a SMRTbell library with the PacBio SMRTbell Template Prep Kit (Pacific Biosciences, Menlo Park, CA, USA) according to the manufacturer’s recommendations. The library was sequenced on five SMRT cells with XL enzyme/C2 chemistry and MagBeads on a PacBio RSII system (Pacific Biosciences, Menlo Park, CA, USA) at 120 min movie length. Sequencing yielded 152,492 filtered subreads corresponding to 420 Mb with a mean subread length of 2753 bases. 50 % of bases were in reads longer than 3400 bases.

### Library preparation for 454 high-throughput sequencing

3-kb and 8-kb mate pair libraries were constructed according to the 454 GS FLX Titanium paired-end protocol with Titanium reagents (Roche) and sequenced on a full PicoTiterPlate on a Genome Sequencer FLX+ Instrument. Sequencing data were processed and bases called using the Roche 454 Software Version 2.6 (shotgun sequencing data processing pipeline).

### Library preparation for Ion Torrent high-throughput sequencing

Short read genome sequencing was performed using the Ion Torrent Personal Genome Machine (Life Technologies, Carlsbad, USA). Following Covaris S2 (Brighton, United Kingdom) fragmentation of 1 μg of genomic DNA, a genomic DNA library was prepared using the Ion Xpress Plus fragment library kit according to the User Guide. Clonal amplification of the resulting library was performed using Ion Sphere particles and emulsion PCR according to the Ion Xpress Template 200 kit manual. The quality of the amplification was estimated, and the sample was loaded onto an Ion 316 chip and sequenced using 125 sequencing cycles according to the Ion Sequencing 200 kit User Guide.

### Library preparation for RNA-seq analysis and Illumina high-throughput sequencing

100 ng of total RNA were used for library preparation according to the instructions provided in the TruSeq Stranded mRNA LT kit (Illumina). An aliquot of the library was analyzed on Qubit (Life Technologies) and Agilent Fragment Analyzer (Advanced Analytical) prior to sequencing on Illumina HiSeq 2000 using the TruSeq SR Cluster Generation Kit v3 and TruSeq SBS Kit v3. Data were processed with the Illumina Pipeline Software v1.82.

### De novo assembly and annotation

Preliminary *de novo* assemblies were done using newbler v2.6 [18] with the 3 and 8 kb 454 paired-ends and using MIRA v3.9.15 [19] with the 454, Ion Torrent and Illumina datasets. Contigs produced by MIRA were aligned onto the scaffolds produced by newbler, and were used to fill or partially fill gaps in these scaffolds and to correct for errors in homopolymeric stretches. Finally the 18b genome was sequenced on PacBio and assembled with HGAP2 [20]. Contigs were aligned to the preliminary assembly and a consensus was made. In case of discrepancies, single nucleotide polymorphisms and 1–2 base-long indels were taken from the newbler’s and MIRA results, while for longer indels the PacBio sequence was considered.

The genome of 18b was annotated using COMPANION [64], which combines the predictions from multiple automated genome annotation engines. We used the predictions from BASys [65], IGS [66], ISGA [67], RAST [68], xBASE [69] and RATT [70], followed by manual curation. We used orthoMCL [24] to infer the orthologs between H37Rv and 18b and removed singleton short hypothetical predictions (<100 aa) from the 18b annotation and manually checked the remaining ortholog groups as well as genes unique to either H37Rv or 18b.

The annotated genome sequence of *M. tuberculosis* 18b and the raw sequence reads were deposited at NCBI (GenBank: CP007299.1, SRA: SRP056193, BioProject: PRJNA236012).

### Phylogeny

Whole genome sequences from *M. tuberculosis* were downloaded from GenBank’s RefSeq Assembly database and aligned against the genome of 18b using LAST v508 [71], converted to mpileup with samtools [72] and SNPs were inferred with VarScan v2.3.7 [73]. Sites with heterozygous variants in a sample (indication of repetitive sequences) were omitted for that sample, as well as all sites corresponding to PE_PGRS genes and insertion sequences. The phylogenetic analysis of the dataset form [21] was done in MEGA6 [74] using the Maximum Likelihood method with the GTR (General Time Reversible) model, since this model had the lowest BIC (Bayesian Information Criterion) score, inferred by MEGA6. The phylogenetic analysis of the large SNP dataset of 1794 *M. tuberculosis* genomes was done in FastTree v2.1.7 [75] using the GTR model and the tree was visualized in Dendroscope 3 [76].

### 3D modelling

IF3N from *M. tuberculosis* 18b was modelled using the webserver Swiss-model [77] using the structures of the N-terminal domain (PDB code: 1TIF) of *G. stearothermophilus* IF3 as template [39]. The structure of the STR-bound 30S ribosomal subunit of *T. thermophilus* (PDB code: 1FJG [38]) was used to analyze the effect of the cytosine insertion in the 16S rRNA of *M. tuberculosis* 18b. For the IF3/30S subunit interaction complex, the approximate orientation of IF3N on the 30S subunit was deduced from hydroxyl-radical cleavage data [40]. Visualization and figures were made using PyMol software (The PyMOL Molecular Graphics System, Version 1.7.4 Schrödinger, LLC.).

### Differential gene expression analysis

Illumina reads were trimmed to remove adapters with flexbar v.2.4 [78] and aligned against the 18b genome sequence with Bowtie2 [79] using default parameters. Feature counting was done with featureCounts from the Subread package v1.4.6 [80]. DESeq2 [81] was used to infer differentially expressed genes. Raw sequence reads were deposited at the GEO database under the accession GSE71066.

For comparison, gene expression data were taken from publications or downloaded from public repositories. When only raw data were available, average expression levels between replicates were considered. Data collected from previous works and used in this study is available in (Additional file 10: Dataset S2). Weighted Venn diagrams were calculated using the R package *venneuler* [82]. Hierarchical gene clustering was done using the R package *gplots*.

### Availability of supporting data

The Genome sequencing project has been deposited at GenBank under the accession PRJNA236012 (http://www.ncbi.nlm.nih.gov/bioproject/236012). The RNA-seq data have been deposited at GEO under the accession GSE71066 (http://www.ncbi.nlm.nih.gov/geo/query/acc.cgi?acc=GSE71066). Other supporting data are included as Additional files.

## Additional files

Additional file 1: Table S1. Novel gene predictions in the genome of 18b. (DOCX 17 kb) Additional file 2: Table S2. InDels larger than 100 bp between the 18b and the H37Rv genomes. (DOCX 32 kb) Additional file 3: Table S3. Genes from H37Rv that are deleted in the genome of 18b. (DOCX 19 kb) Additional file 4: Table S4. Genes from 18b that are deleted in the genome of H37Rv. (DOCX 16 kb) Additional file 5: Table S5. Protein coding genes that are annotated as functional in H37Rv and as pseudogenes in 18b. (DOCX 21 kb) Additional file 6: Table S6. Genes in the genome of 18b that span two genes in H37Rv. (DOCX 24 kb) Additional file 7: Table S7. Positions of IS6*110* in the genome sequences of *M. tuberculosis* 18b and H37Rv. (DOCX 27 kb) Additional file 8: Table S8. SNPs unique to *M. tuberculosis* 18b from a comparison with 1793 *M. tuberculosis* genomes. (DOCX 25 kb) Additional file 9: Dataset S1. Occurrence of the 66 unique 18b SNPs in the SRA. (XLSX 2826 kb) Additional file 10: Dataset S2. Differential gene expression analysis results of SS18b (2 weeks STR depletion, 4 weeks STR depletion and re-addition of STR). (XLSX 2308 kb) Additional file 11: Figure S1. Weighted Venn diagrams representing overlap of the differential gene expression results from this work with those from stationary phase induction and response to macrophage infection. (PDF 163 kb) Additional file 12: Figure S2. Hierarchical clustering of differentially expressed genes in SS18b and from previous works showing >2-fold differences. (PDF 332 kb) Additional file 13: Figure S3. Hierarchical clustering of differentially expressed genes in SS18b and the results from previous works with 2-fold differences displayed as shrunk and expanded images. (PDF 748 kb) Additional file 14: Figure S4. Hierarchical clustering of differentially expressed genes in SS18b and previous works. Expanded version of Fig. 4 including gene names. (PDF 239 kb)

## Abbreviations

STR: streptomycin
SS18b: streptomycin-starved 18b
IF3: initiation factor 3
FDR: false discovery rate

## References

[1] Global tuberculosis report 2014. [http://www.who.int/tb/publications/global_report/en/].

[2] Dye C, Scheele S, Dolin P, Pathania V, Raviglione MC (1999). Consensus statement. Global burden of tuberculosis: estimated incidence, prevalence, and mortality by country. WHO Global Surveillance and Monitoring Project. JAMA.

[3] Dutta NK, Karakousis PC (2014). Latent tuberculosis infection: myths, models, and molecular mechanisms. Microbiol Mol Biol Rev.

[4] Betts JC, Lukey PT, Robb LC, McAdam RA, Duncan K (2002). Evaluation of a nutrient starvation model of Mycobacterium tuberculosis persistence by gene and protein expression profiling. Mol Microbiol.

[5] Hampshire T, Soneji S, Bacon J, James BW, Hinds J, Laing K, Stabler RA, Marsh PD, Butcher PD (2004). Stationary phase gene expression of Mycobacterium tuberculosis following a progressive nutrient depletion: a model for persistent organisms?. Tuberc Edinb Scotl.

[6] Rifat D, Bishai WR, Karakousis PC (2009). Phosphate depletion: a novel trigger for mycobacterium tuberculosis persistence. J Infect Dis.

[7] Wayne LG, Hayes LG (1996). An in vitro model for sequential study of shiftdown of Mycobacterium tuberculosis through two stages of nonreplicating persistence. Infect Immun.

[8] Voskuil MI, Schnappinger D, Visconti KC, Harrell MI, Dolganov GM, Sherman DR, Schoolnik GK (2003). Inhibition of respiration by nitric oxide induces a Mycobacterium tuberculosis dormancy program. J Exp Med.

[9] Deb C, Lee C-M, Dubey VS, Daniel J, Abomoelak B, Sirakova TD, Pawar S, Rogers L, Kolattukudy PE (2009). A novel in vitro multiple-stress dormancy model for mycobacterium tuberculosis generates a lipid-loaded, drug-tolerant, dormant pathogen. PLoS ONE.

[10] Hashimoto T (1955). Experimental studies on the mechanism of infection and immunity in tuberculosis from the analytical standpoint of streptomycin-dependent tubercle bacilli. 1. Isolation and biological characteristics of a streptomycin-dependent mutant, and effect of streptomycin administration on its pathogenicity in guinea-pigs. Kekkaku.

[11] Sala C, Dhar N, Hartkoorn RC, Zhang M, Ha YH, Schneider P, Cole ST (2010). Simple model for testing drugs against nonreplicating mycobacterium tuberculosis. Antimicrob Agents Chemother.

[12] Zhang M, Sala C, Hartkoorn RC, Dhar N, Mendoza-Losana A, Cole ST (2012). Streptomycin-starved Mycobacterium tuberculosis 18b, a drug discovery tool for latent tuberculosis. Antimicrob Agents Chemother.

[13] Zhang M, Sala C, Dhar N, Vocat A, Sambandamurthy VK, Sharma S, Marriner G, Balasubramanian V, Cole ST (2014). In vitro and in vivo activities of three oxazolidinones against nonreplicating Mycobacterium tuberculosis. Antimicrob Agents Chemother.

[14] Vocat A, Hartkoorn RC, Lechartier B, Zhang M, Dhar N, Cole ST, Sala C (2015). Bioluminescence for assessing drug potency against nonreplicating mycobacterium tuberculosis. Antimicrob Agents Chemother.

[15] Kashino SS, Napolitano DR, Skobe Z, Campos-Neto A (2008). Guinea pig model of Mycobacterium tuberculosis latent/dormant infection. Microbes Infect Inst Pasteur.

[16] Kashino SS, Ovendale P, Izzo A, Campos-Neto A (2006). Unique model of dormant infection for tuberculosis vaccine development. Clin Vaccine Immunol.

[17] Mishra BB, Rathinam VAK, Martens GW, Martinot AJ, Kornfeld H, Fitzgerald KA, Sassetti CM (2013). Nitric oxide controls the immunopathology of tuberculosis by inhibiting NLRP3 inflammasome-dependent processing of IL-1β. Nat Immunol.

[18] Margulies M, Egholm M, Altman WE, Attiya S, Bader JS, Bemben LA, Berka J, Braverman MS, Chen Y-J, Chen Z, Dewell SB, Du L, Fierro JM, Gomes XV, Godwin BC, He W, Helgesen S, Ho CH, Ho CH, Irzyk GP, Jando SC, Alenquer MLI, Jarvie TP, Jirage KB, Kim J-B, Knight JR, Lanza JR, Leamon JH, Lefkowitz SM, Lei M (2005). Genome sequencing in microfabricated high-density picolitre reactors. Nature.

[19] Chevreux B, Wetter T, Suhai S (1999). Genome sequence assembly using trace signals and additional sequence information. Computer science and biology: proceedings of the German conference on bioinformatics (GCB). Volume 99.

[20] Chin C-S, Alexander DH, Marks P, Klammer AA, Drake J, Heiner C, Clum A, Copeland A, Huddleston J, Eichler EE, Turner SW, Korlach J (2013). Nonhybrid, finished microbial genome assemblies from long-read SMRT sequencing data. Nat Methods.

[21] Merker M, Blin C, Mona S, Duforet-Frebourg N, Lecher S, Willery E, Blum M, Rüsch-Gerdes S, Mokrousov I, Aleksic E, Allix-Béguec C, Antierens A, Augustynowicz-Kopeć E, Ballif M, Barletta F, Beck HP, Barry Iii CE, Bonnet M, Borroni E, Campos-Herrero I, Cirillo D, Cox H, Crowe S, Crudu V, Diel R, Drobniewski F, Fauville-Dufaux M, Gagneux S, Ghebremichael S, Hanekom M, et al. Evolutionary history and global spread of the Mycobacterium tuberculosis Beijing lineage. Nat Genet. 2015, advance online publication. 10.1038/ng.3195 PMC11044984 25599400

[22] Coll F, McNerney R, Guerra-Assunção JA, Glynn JR, Perdigão J, Viveiros M, Portugal I, Pain A, Martin N, Clark TG (2014). A robust SNP barcode for typing Mycobacterium tuberculosis complex strains. Nat Commun.

[23] Coll F, Preston M, Guerra-Assunção JA, Hill-Cawthorn G, Harris D, Perdigão J, Viveiros M, Portugal I, Drobniewski F, Gagneux S, Glynn JR, Pain A, Parkhill J, McNerney R, Martin N, Clark TG (2014). PolyTB: a genomic variation map for Mycobacterium tuberculosis. Tuberc Edinb Scotl.

[24] Li L, Stoeckert CJ, Roos DS (2003). OrthoMCL: identification of ortholog groups for eukaryotic genomes. Genome Res.

[25] Schubert OT, Mouritsen J, Ludwig C, Röst HL, Rosenberger G, Arthur PK, Claassen M, Campbell DS, Sun Z, Farrah T, Gengenbacher M, Maiolica A, Kaufmann SHE, Moritz RL, Aebersold R (2013). The Mtb proteome library: a resource of assays to quantify the complete proteome of Mycobacterium tuberculosis. Cell Host Microbe.

[26] Dale JW, Brittain D, Cataldi AA, Cousins D, Crawford JT, Driscoll J, Heersma H, Lillebaek T, Quitugua T, Rastogi N, Skuce RA, Sola C, Van Soolingen D, Vincent V (2001). Spacer oligonucleotide typing of bacteria of the Mycobacterium tuberculosis complex: recommendations for standardised nomenclature. Int J Tuberc Lung Dis Off J Int Union Tuberc Lung Dis.

[27] Målen H, Berven FS, Fladmark KE, Wiker HG (2007). Comprehensive analysis of exported proteins from Mycobacterium tuberculosis H37Rv. Proteomics.

[28] Kruh NA, Troudt J, Izzo A, Prenni J, Dobos KM (2010). Portrait of a pathogen: the Mycobacterium tuberculosis proteome in vivo. PLoS One.

[29] Fallow A, Domenech P, Reed MB (2010). Strains of the East Asian (W/Beijing) lineage of Mycobacterium tuberculosis are DosS/DosT-DosR two-component regulatory system natural mutants. J Bacteriol.

[30] Muñoz-Elías EJ, McKinney JD (2005). Mycobacterium tuberculosis isocitrate lyases 1 and 2 are jointly required for in vivo growth and virulence. Nat Med.

[31] Reed MB, Domenech P, Manca C, Su H, Barczak AK, Kreiswirth BN (2004). A glycolipid of hypervirulent tuberculosis strains that inhibits the innate immune response. Nature.

[32] Ribeiro SCM, Gomes LL, Amaral EP, Andrade MRM, Almeida FM, Rezende AL, Lanes VR, Carvalho ECQ, Suffys PN, Mokrousov I, Lasunskaia EB (2014). Mycobacterium tuberculosis strains of the modern sublineage of the Beijing Family are more likely to display increased virulence than strains of the ancient sublineage. J Clin Microbiol.

[33] Manabe YC, Dannenberg AM, Tyagi SK, Hatem CL, Yoder M, Woolwine SC, Zook BC, Pitt MLM, Bishai WR (2003). Different strains of mycobacterium tuberculosis cause various spectrums of disease in the rabbit model of tuberculosis. Infect Immun.

[34] Coscolla M, Gagneux S (2014). Consequences of genomic diversity in Mycobacterium tuberculosis. Semin Immunol.

[35] Gordon SV, Heym B, Parkhill J, Barrell B, Cole ST (1999). New insertion sequences and a novel repeated sequence in the genome of Mycobacterium tuberculosis H37Rv. Microbiol Read Engl.

[36] Kremer K, Glynn JR, Lillebaek T, Niemann S, Kurepina NE, Kreiswirth BN (2004). Definition of the Beijing/W Lineage of Mycobacterium tuberculosis on the Basis of Genetic Markers. J Clin Microbiol.

[37] Honoré N, Marchal G, Cole ST (1995). Novel mutation in 16S rRNA associated with streptomycin dependence in Mycobacterium tuberculosis. Antimicrob Agents Chemother.

[38] Carter AP, Clemons WM, Brodersen DE, Morgan-Warren RJ, Wimberly BT, Ramakrishnan V (2000). Functional insights from the structure of the 30S ribosomal subunit and its interactions with antibiotics. Nature.

[39] Biou V, Shu F, Ramakrishnan V (1995). X-ray crystallography shows that translational initiation factor IF3 consists of two compact alpha/beta domains linked by an alpha-helix. EMBO J.

[40] Dallas A, Noller HF (2001). Interaction of translation initiation factor 3 with the 30S ribosomal subunit. Mol Cell.

[41] Moore PB (2013). Ribosomal ambiguity made less ambiguous. Proc Natl Acad Sci U S A.

[42] Gualerzi CO, Pon CL (1990). Initiation of mRNA translation in prokaryotes. Biochemistry (Mosc).

[43] Bruhns J, Gualerzi C (1980). Structure--function relationship in Escherichia coli initiation factors: role of tyrosine residues in ribosomal binding and functional activity of IF-3. Biochemistry (Mosc).

[44] Pioletti M, Schlünzen F, Harms J, Zarivach R, Glühmann M, Avila H, Bashan A, Bartels H, Auerbach T, Jacobi C, Hartsch T, Yonath A, Franceschi F (2001). Crystal structures of complexes of the small ribosomal subunit with tetracycline, edeine and IF3. EMBO J.

[45] Manganelli R, Voskuil MI, Schoolnik GK, Smith I (2001). The Mycobacterium tuberculosis ECF sigma factor sigmaE: role in global gene expression and survival in macrophages. Mol Microbiol.

[46] Homolka S, Niemann S, Russell DG, Rohde KH. Functional genetic diversity among mycobacterium tuberculosis complex clinical isolates: delineation of conserved core and lineage-specific transcriptomes during intracellular survival. PLoS Pathog. 2010;6. 10.1371/journal.ppat.1000988 PMC2900310 20628579

[47] Voskuil MI, Visconti KC, Schoolnik GK (2004). Mycobacterium tuberculosis gene expression during adaptation to stationary phase and low-oxygen dormancy. Tuberc Edinb Scotl.

[48] Bacon J, James BW, Wernisch L, Williams A, Morley KA, Hatch GJ, Mangan JA, Hinds J, Stoker NG, Butcher PD, Marsh PD (2004). The influence of reduced oxygen availability on pathogenicity and gene expression in Mycobacterium tuberculosis. Tuberc Edinb Scotl.

[49] Muttucumaru DGN, Roberts G, Hinds J, Stabler RA, Parish T (2004). Gene expression profile of Mycobacterium tuberculosis in a non-replicating state. Tuberc Edinb Scotl.

[50] Boshoff HIM, Myers TG, Copp BR, McNeil MR, Wilson MA, Barry CE (2004). The transcriptional responses of Mycobacterium tuberculosis to inhibitors of metabolism: novel insights into drug mechanisms of action. J Biol Chem.

[51] Hu Y, Coates AR (1999). Transcription of two sigma 70 homologue genes, sigA and sigB, in stationary-phase Mycobacterium tuberculosis. J Bacteriol.

[52] Murphy DJ, Brown JR (2007). Identification of gene targets against dormant phase Mycobacterium tuberculosis infections. BMC Infect Dis.

[53] Rengarajan J, Bloom BR, Rubin EJ (2005). Genome-wide requirements for Mycobacterium tuberculosis adaptation and survival in macrophages. Proc Natl Acad Sci U S A.

[54] Sassetti CM, Boyd DH, Rubin EJ (2003). Genes required for mycobacterial growth defined by high density mutagenesis. Mol Microbiol.

[55] DiChiara JM, Contreras-Martinez LM, Livny J, Smith D, McDonough KA, Belfort M (2010). Multiple small RNAs identified in Mycobacterium bovis BCG are also expressed in Mycobacterium tuberculosis and Mycobacterium smegmatis. Nucleic Acids Res.

[56] Arnvig KB, Comas I, Thomson NR, Houghton J, Boshoff HI, Croucher NJ, Rose G, Perkins TT, Parkhill J, Dougan G, Young DB (2011). Sequence-based analysis uncovers an abundance of non-coding RNA in the total transcriptome of Mycobacterium tuberculosis. PLoS Pathog.

[57] Arnvig K, Young D (2012). Non-coding RNA and its potential role in Mycobacterium tuberculosis pathogenesis. RNA Biol.

[58] Uplekar S, Rougemont J, Cole ST, Sala C (2013). High-resolution transcriptome and genome-wide dynamics of RNA polymerase and NusA in Mycobacterium tuberculosis. Nucleic Acids Res.

[59] Rustad TR, Harrell MI, Liao R, Sherman DR (2008). The enduring hypoxic response of Mycobacterium tuberculosis. PLoS One.

[60] Ohno H, Zhu G, Mohan VP, Chu D, Kohno S, Jacobs WR, Chan J (2003). The effects of reactive nitrogen intermediates on gene expression in Mycobacterium tuberculosis. Cell Microbiol.

[61] Kendall SL, Movahedzadeh F, Rison SCG, Wernisch L, Parish T, Duncan K, Betts JC, Stoker NG (2004). The Mycobacterium tuberculosis dosRS two-component system is induced by multiple stresses. Tuberculosis.

[62] Zhou L, Yang L, Zeng X, Danzheng J, Zheng Q, Liu J, Liu F, Xin Y, Cheng X, Su M, Ma Y, Hao X (2015). Transcriptional and proteomic analyses of two-component response regulators in multidrug-resistant Mycobacterium tuberculosis. Int J Antimicrob Agents.

[63] Rand L, Hinds J, Springer B, Sander P, Buxton RS, Davis EO (2003). The majority of inducible DNA repair genes in Mycobacterium tuberculosis are induced independently of RecA. Mol Microbiol.

[64] Ederveen THA, Overmars L, van Hijum SAFT (2013). Reduce manual curation by combining gene predictions from multiple annotation engines, a case study of start codon prediction. PLoS ONE.

[65] Van Domselaar GH, Stothard P, Shrivastava S, Cruz JA, Guo A, Dong X, Lu P, Szafron D, Greiner R, Wishart DS (2005). BASys: a web server for automated bacterial genome annotation. Nucleic Acids Res.

[66] Galens K, Orvis J, Daugherty S, Creasy HH, Angiuoli S, White O, Wortman J, Mahurkar A, Giglio MG (2011). The IGS standard operating procedure for automated prokaryotic annotation. Stand Genomic Sci.

[67] Hemmerich C, Buechlein A, Podicheti R, Revanna KV, Dong Q (2010). An Ergatis-based prokaryotic genome annotation web server. Bioinforma Oxf Engl.

[68] Aziz RK, Bartels D, Best AA, DeJongh M, Disz T, Edwards RA, Formsma K, Gerdes S, Glass EM, Kubal M, Meyer F, Olsen GJ, Olson R, Osterman AL, Overbeek RA, McNeil LK, Paarmann D, Paczian T, Parrello B, Pusch GD, Reich C, Stevens R, Vassieva O, Vonstein V, Wilke A, Zagnitko O (2008). The RAST Server: Rapid Annotations using Subsystems Technology. BMC Genomics.

[69] Chaudhuri RR, Loman NJ, Snyder LAS, Bailey CM, Stekel DJ, Pallen MJ (2008). xBASE2: a comprehensive resource for comparative bacterial genomics. Nucleic Acids Res.

[70] Otto TD, Dillon GP, Degrave WS, Berriman M (2011). RATT: Rapid Annotation Transfer Tool. Nucleic Acids Res.

[71] Kiełbasa SM, Wan R, Sato K, Horton P, Frith MC (2011). Adaptive seeds tame genomic sequence comparison. Genome Res.

[72] Li H, Handsaker B, Wysoker A, Fennell T, Ruan J, Homer N, Marth G, Abecasis G, Durbin R (2009). The sequence Alignment/Map format and SAMtools. Bioinformatics.

[73] Koboldt DC, Zhang Q, Larson DE, Shen D, McLellan MD, Lin L, Miller CA, Mardis ER, Ding L, Wilson RK (2012). VarScan 2: somatic mutation and copy number alteration discovery in cancer by exome sequencing. Genome Res.

[74] Tamura K, Stecher G, Peterson D, Filipski A, Kumar S (2013). MEGA6: Molecular Evolutionary Genetics Analysis Version 6.0. Mol Biol Evol.

[75] Price MN, Dehal PS, Arkin AP (2010). FastTree 2 – approximately maximum-likelihood trees for large alignments. PLoS ONE.

[76] Huson DH, Scornavacca C (2012). Dendroscope 3: an interactive tool for rooted phylogenetic trees and networks. Syst Biol.

[77] Arnold K, Bordoli L, Kopp J, Schwede T (2006). The SWISS-MODEL workspace: a web-based environment for protein structure homology modelling. Bioinformatics.

[78] Dodt M, Roehr JT, Ahmed R, Dieterich C (2012). FLEXBAR—flexible barcode and adapter processing for next-generation sequencing platforms. Biology.

[79] Langmead B, Salzberg SL (2012). Fast gapped-read alignment with Bowtie 2. Nat Methods.

[80] Liao Y, Smyth GK, Shi W (2013). FeatureCounts: an efficient general purpose program for assigning sequence reads to genomic features. Bioinformatics.

[81] Love MI, Huber W, Anders S (2014). Moderated estimation of fold change and dispersion for RNA-seq data with DESeq2. Genome Biol.

[82] Wilkinson L (2012). Exact and approximate area-proportional circular Venn and Euler diagrams. IEEE Trans Vis Comput Graph.

