# Supplementary material for: Genomic and transcriptomic analysis of the streptomycin-dependent Mycobacterium tuberculosis strain 18b
Source: BMC Genomics. 2016 Mar 5;17:190. doi: 10.1186/s12864-016-2528-2 (PMC4779234; doi:10.1186/s12864-016-2528-2)
Supplement: Additional file 1: Table S1. — Novel gene predictions in the genome of 18b. (DOCX 17 kb) [file 12864_2016_2528_MOESM1_ESM.docx]

Table S1: Novel gene predictions in the genome of 18b.

| Locus tag | Start | End | Strand | Feature | Length | Gene product | Note |
| --- | --- | --- | --- | --- | --- | --- | --- |
| MT18B_0294 | 273119 | 273313 | - | CDS | 195 | hypothetical protein | Identified by proteomics*. |
| MT18B_0559 | 543131 | 543454 | - | CDS | 324 | hypothetical protein | Supported by RNA-seq**. |
| MT18B_0715 | 663889 | 664056 | - | CDS | 168 | hypothetical protein | Identified by proteomics. |
| MT18B_0770 | 702922 | 703119 | - | CDS | 198 | hypothetical protein | Identified by proteomics. |
| MT18B_0895 | 798140 | 798733 | - | CDS | 594 | hypothetical protein | Identified in *M. caprae.* |
| MT18B_0960 | 834150 | 834296 | - | pseudogene | 147 | possible 3-hydroxyisobutyrate dehydrogenase (fragment) | Duplicated and degraded fragment of MT18B_0975/ Rv0751c |
| MT18B_1307 | 1112038 | 1112571 | - | pseudogene | 534 | methyltransferase |  |
| MT18B_1534 | 1280014 | 1280202 | + | CDS | 189 | hypothetical protein | Identified by proteomics. |
| MT18B_2085 | 1789634 | 1790398 | - | CDS | 765 | hypothetical protein | Supported by RNA-seq. |
| MT18B_2742 | 2333806 | 2334249 | + | CDS | 444 | hypothetical protein | Supported by RNA-seq. |
| MT18B_3040 | 2572032 | 2572364 | + | CDS | 333 | hypothetical protein | Supported by RNA-seq. |
| MT18B_3159 | 2673107 | 2673316 | + | CDS | 210 | hypothetical protein | Identified by proteomics. |
| MT18B_3509 | 2959695 | 2960117 | + | CDS | 423 | hypothetical protein | Supported by RNA-seq. |
| MT18B_3634 | 3046196 | 3046600 | + | CDS | 405 | hypothetical protein | Identified by proteomics. Supported by RNA-seq. |
| MT18B_3780 | 3153149 | 3153481 | + | CDS | 333 | hypothetical protein | Supported by RNA-seq. |
| MT18B_3906 | 3288620 | 3288937 | + | CDS | 318 | hypothetical protein | Identified in *M. caprae.* |
| MT18B_3944 | 3317857 | 3318051 | + | CDS | 195 | 50S ribosomal protein L28 RpmB3 | Should be annotated in Rv too. Supported with RNA-seq and proteomics |
| MT18B_3976 | 3343370 | 3343672 | + | CDS | 303 | hypothetical protein | Supported by RNA-seq. |
| MT18B_5307 | 3373184 | 3373348 | + | CDS | 165 | hypothetical protein | Identified by proteomics. Supported by RNA-seq. |
| MT18B_4104 | 3445300 | 3445602 | - | CDS | 303 | hypothetical protein | Supported by RNA-seq. |
| MT18B_4276 | 3575812 | 3576141 | - | CDS | 330 | hypothetical protein | Annotated as putative helicase in some *Mtb* strains. No RNA-seq coverage. |

*Schubert et al. 2013.

**RNA-seq data from this work.
