# Supplementary material for: Genomic and transcriptomic analysis of the streptomycin-dependent Mycobacterium tuberculosis strain 18b
Source: BMC Genomics. 2016 Mar 5;17:190. doi: 10.1186/s12864-016-2528-2 (PMC4779234; doi:10.1186/s12864-016-2528-2)
Supplement: Additional file 2: Table S2. — InDels larger than 100 bp between the 18b and the H37Rv genomes. (DOCX 32 kb) [file 12864_2016_2528_MOESM2_ESM.docx]

Table S2: InDels larger than 100 bp between the 18b and the H37Rv genomes. Genes in red are partially deleted at their 5', genes in green are partially deleted at their 3' and genes in blue have an internal partial deletion.

| 18b | H37Rv | Length (bp) | 18b start | 18b end | H37Rv start | H37Rv end | Deleted genes | Gene product |
| --- | --- | --- | --- | --- | --- | --- | --- | --- |
| In | Del | 6'356 | 2'257'898 | 2'264'254 | 2'268'725 | - | MT18B_2662 | putative transposase |
|  |  |  |  |  |  |  | MT18B_2663 | transposase |
|  |  |  |  |  |  |  | MT18B_2665 | hypothetical protein |
|  |  |  |  |  |  |  | MT18B_2666 | hypothetical protein |
| In | Del | 4'493 | 2'202'739 | 2'207'232 | 2'219'418 | - | MT18B_2574 | PE family protein |
|  |  |  |  |  |  |  | MT18B_2577 | hypothetical protein |
|  |  |  |  |  |  |  | MT18B_2578 | hypothetical protein |
| In | Del | 3'571 | 3'699'874 | 3'703'445 | 3'710'381 | - | MT18B_4414 | pterin-4-alpha-carbinolamine dehydratase |
|  |  |  |  |  |  |  | MT18B_4415 | molybdenum cofactor biosynthesis protein subunit MoaA |
|  |  |  |  |  |  |  | MT18B_4420 | hypothetical protein |
| In | Del | 3'496 | 3'726'087 | 3'729'583 | 3'732'792 | - | MT18B_4446 | PPE family protein |
| In | Del | 2'428 | 3'845'258 | 3'847'686 | 3'846'562 | - | MT18B_4564 | PPE family protein |
|  |  |  |  |  |  |  | MT18B_4565 | PPE family protein |
|  |  |  |  |  |  |  | MT18B_5287 | transposase (fragment) |
| In | Del | 2'151 | 3'519'506 | 3'521'657 | 3'529'285 | - | MT18B_4204 | PPE family protein |
| In | Del | 1'861 | 2'147'445 | 2'149'306 | 2'165'988 | - | MT18B_2494 | transposase |
|  |  |  |  |  |  |  | MT18B_2495 | putative transposase |
| In | Del | 1'682 | 1'480'341 | 1'482'023 | 1'482'525 | - | MT18B_1746 | adenylate cyclase |
| In | Del | 1'358 | 3'481'745 | 3'483'103 | 3'494'213 | - | MT18B_4156 | transposase |
|  |  |  |  |  |  |  | MT18B_4158 | putative transposase |
| In | Del | 1'357 | 3'842'028 | 3'843'385 | 3'844'681 | - | MT18B_4559 | transposase |
|  |  |  |  |  |  |  | MT18B_4561 | transposase |
| In | Del | 1'356 | 1'595 | 2'951 | 1'594 | - | MT18B_0002 | putative transposase |
|  |  |  |  |  |  |  | MT18B_0003 | transposase |
| In | Del | 1'356 | 2'251'442 | 2'252'798 | 2'263'627 | - | MT18B_2648 | transposase |
|  |  |  |  |  |  |  | MT18B_2651 | putative transposase |
| In | Del | 1'356 | 3'364'672 | 3'366'028 | 3'378'553 | - | MT18B_4006 | transposase |
|  |  |  |  |  |  |  | MT18B_4007 | putative transposase |
| In | Del | 1'352 | 3'105'242 | 3'106'594 | 3'115'064 | - | MT18B_3720 | putative transposase |
|  |  |  |  |  |  |  | MT18B_3721 | transposase |
| In | Del | 1'335 | 3'490'556 | 3'491'891 | 3'501'665 | - | MT18B_4173 | PPE family protein |
| In | Del | 954 | 1'656'061 | 1'657'015 | 1'657'017 | - | MT18B_1936 | transposase |
|  |  |  |  |  |  |  | MT18B_1937 | putative transposase |
| In | Del | 950 | 2'583'235 | 2'584'185 | 2'592'358 | - | MT18B_3061 | universal stress protein |
|  |  |  |  |  |  |  | MT18B_3062 | periplasmic sugar-binding lipoprotein uspc |
| In | Del | 798 | 2'628'780 | 2'629'578 | 2'635'576 | - | MT18B_3115 | PPE family protein |
| In | Del | 614 | 3'218'252 | 3'218'866 | 3'232'866 | - |  |  |
| In | Del | 554 | 4'052'904 | 4'053'458 | 4'053'549 | - | MT18B_4785 | hypothetical protein |
|  |  |  |  |  |  |  | MT18B_4786 | hypothetical protein |
| In | Del | 532 | 741'082 | 741'614 | 742'635 | - | MT18B_0824 | alpha-mannosidase |
| In | Del | 407 | 3'817'529 | 3'817'936 | 3'820'589 | - |  |  |
| In | Del | 397 | 4'120'980 | 4'121'377 | 4'121'053 | - | MT18B_4880 | hypothetical protein |
| In | Del | 278 | 40'409 | 40'687 | 39'069 | - |  |  |
| In | Del | 229 | 3'704'801 | 3'705'030 | 3'711'736 | - |  |  |
| In | Del | 207 | 1'632'170 | 1'632'377 | 1'633'538 | - | MT18B_1910 | PE_PGRS family protein |
| In | Del | 202 | 2'986'151 | 2'986'353 | 2'996'168 | - |  |  |
| In | Del | 192 | 40'409 | 40'601 | 39'069 | - |  |  |
| In | Del | 189 | 1'634'981 | 1'635'170 | 1'636'169 | - | MT18B_1915 | PE_PGRS family protein |
| In | Del | 160 | 149'147 | 149'307 | 150'903 | - | MT18B_0169 | PE_PGRS family protein |
| In | Del | 116 | 4'157'270 | 4'157'386 | 4'156'967 | - |  |  |
| In | Del | 114 | 2'396'232 | 2'396'346 | 2'401'927 | - |  |  |
| Del | In | 9'245 | 1'779'680 | - | 1'779'278 | 1'788'523 | Rv1573 | Probable PhiRv1 phage protein |
|  |  |  |  |  |  |  | Rv1574 | Probable PhiRv1 phage related protein |
|  |  |  |  |  |  |  | Rv1575 | Probable PhiRv1 phage protein |
|  |  |  |  |  |  |  | Rv1576c | Probable PhiRv1 phage protein |
|  |  |  |  |  |  |  | Rv1577c | Probable PhiRv1 phage protein |
|  |  |  |  |  |  |  | Rv1578c | Probable PhiRv1 phage protein |
|  |  |  |  |  |  |  | Rv1579c | Probable PhiRv1 phage protein |
|  |  |  |  |  |  |  | Rv1580c | Probable PhiRv1 phage protein |
|  |  |  |  |  |  |  | Rv1581c | Probable PhiRv1 phage protein |
|  |  |  |  |  |  |  | Rv1582c | Probable PhiRv1 phage protein |
|  |  |  |  |  |  |  | Rv1583c | Probable PhiRv1 phage protein |
|  |  |  |  |  |  |  | Rv1584c | Possible PhiRv1 phage protein |
|  |  |  |  |  |  |  | Rv1585c | Possible phage PhiRv1 protein |
|  |  |  |  |  |  |  | Rv1586c | Probable PhiRv1 integrase |
|  |  |  |  |  |  |  | Rv1587c | Partial REP13E12 repeat protein |
| Del | In | 6'049 | 3'113'202 | - | 3'121'880 | 3'127'929 | Rv2816c | Conserved hypothetical protein |
|  |  |  |  |  |  |  | Rv2817c | Conserved hypothetical protein |
|  |  |  |  |  |  |  | Rv2818c | Hypothetical protein |
|  |  |  |  |  |  |  | Rv2819c | Hypothetical protein |
|  |  |  |  |  |  |  | Rv2820c | Hypothetical protein |
| Del | In | 3'470 | 81'260 | - | 79'565 | 83'035 | Rv0071 | Possible maturase |
|  |  |  |  |  |  |  | Rv0072 | Probable glutamine-transport transmembrane protein ABC transporter |
|  |  |  |  |  |  |  | Rv0073 | Probable glutamine-transport ATP-binding protein ABC transporter |
|  |  |  |  |  |  |  | Rv0074 | Conserved protein |
| Del | In | 1'358 | 2'361'031 | - | 2'365'424 | 2'366'782 | Rv2105 | Putative transposase for insertion sequence element IS6110 (fragment) |
|  |  |  |  |  |  |  | Rv2106 | Probable transposase |
| Del | In | 1'357 | 888'208 | - | 889'021 | 890'378 | Rv0795 | Putative transposase for insertion sequence element IS6110 (fragment) |
|  |  |  |  |  |  |  | Rv0796 | Putative transposase for insertion sequence element IS6110 |
| Del | In | 1'356 | 2'424'540 | - | 2'430'117 | 2'431'473 | Rv2167c | Probable transposase |
|  |  |  |  |  |  |  | Rv2168c | Putative transposase for insertion sequence element IS6110 (fragment) |
| Del | In | 1'356 | 2'542'256 | - | 2'550'014 | 2'551'370 | Rv2278 | Putative transposase for insertion sequence element IS6110 (fragment) |
|  |  |  |  |  |  |  | Rv2279 | Probable transposase |
| Del | In | 1'356 | 2'777'265 | - | 2'784'617 | 2'785'973 | Rv2479c | Probable transposase |
|  |  |  |  |  |  |  | Rv2480c | Possible transposase for insertion sequence element IS6110 (fragment) |
| Del | In | 1'356 | 2'963'393 | - | 2'972'109 | 2'973'465 | Rv2648 | Probable transposase for insertion sequence element IS6110 (fragment) |
|  |  |  |  |  |  |  | Rv2649 | Probable transposase for insertion sequence element IS6110 |
| Del | In | 1'356 | 3'543'602 | - | 3'551'230 | 3'552'586 | Rv3184 | Probable transposase for insertion sequence element IS6110 (fragment) |
|  |  |  |  |  |  |  | Rv3185 | Probable transposase |
| Del | In | 1'356 | 3'543'727 | - | 3'552'713 | 3'554'069 | Rv3186 | Probable transposase for insertion sequence element IS6110 (fragment) |
|  |  |  |  |  |  |  | Rv3187 | Probable transposase |
| Del | In | 1'356 | 3'891'959 | - | 3'890'779 | 3'892'135 | Rv3474 | Possible transposase for insertion element IS6110 (fragment) |
|  |  |  |  |  |  |  | Rv3475 | Possible transposase for insertion element IS6110 [second part] |
| Del | In | 1'353 | 1'978'472 | 1'979'825 | 1'986'639 | 1'998'622 | Rv1755c | Probable phospholipase C 4 (fragment) PlcD |
|  |  |  |  |  |  |  | Rv1758 | Probable cutinase Cut1 |
|  |  |  |  |  |  |  | Rv1759c | PE-PGRS family protein Wag22 |
|  |  |  |  |  |  |  | Rv1760 | Possible triacylglycerol synthase (diacylglycerol acyltransferase) |
|  |  |  |  |  |  |  | Rv1761c | Possible exported protein |
|  |  |  |  |  |  |  | Rv1762c | Unknown protein |
|  |  |  |  |  |  |  | Rv1763 | Putative transposase for insertion sequence element IS6110 (fragment) |
|  |  |  |  |  |  |  | Rv1764 | Putative transposase |
|  |  |  |  |  |  |  | Rv1765c | Conserved hypothetical protein |
| Del | In | 1'155 | 1'541'414 | - | 1'542'670 | 1'543'825 |  |  |
| Del | In | 709 | 2'528'383 | - | 2'535'432 | 2'536'141 | Rv2263 | Possible oxidoreductase |
| Del | In | 274 | 3'111'790 | - | 3'120'249 | 3'120'523 |  |  |
| Del | In | 144 | 1'542'568 | - | 1'543'825 | 1'543'969 | Rv1369c | Probable transposase |
|  |  |  |  |  |  |  | Rv1371 | Probable conserved membrane protein |
| Del | In | 126 | 839'420 | - | 840'097 | 840'223 |  |  |
| Del | In | 113 | 1'449'819 | - | 1'451'882 | 1'451'995 |  |  |
