# Supplementary material for: Genomic and transcriptomic analysis of the streptomycin-dependent Mycobacterium tuberculosis strain 18b
Source: BMC Genomics. 2016 Mar 5;17:190. doi: 10.1186/s12864-016-2528-2 (PMC4779234; doi:10.1186/s12864-016-2528-2)
Supplement: Additional file 3: Table S3. — Genes from H37Rv that are deleted in the genome of 18b. (DOCX 19 kb) [file 12864_2016_2528_MOESM3_ESM.docx]

Table S3: Genes from the genome of H37Rv that are deleted in the genome of 18b.

| Gene | Product | Functional category | Note |
| --- | --- | --- | --- |
| Rv0071 | Possible maturase | Information pathways | Deleted in 18b |
| Rv0072 | Probable glutamine-transport transmembrane protein ABC transporter | Cell wall and cell processes | Deleted in 18b |
| Rv0073 | Probable glutamine-transport ATP-binding protein ABC transporter | Cell wall and cell processes | Deleted in 18b |
| Rv0795 | Putative transposase for insertion sequence element IS6110 (fragment) | Insertion seqs and phages | Deleted in 18b |
| Rv0796 | Putative transposase for insertion sequence element IS6110 | Insertion seqs and phages | Deleted in 18b |
| Rv1369c | Probable transposase | Insertion seqs and phages | Deleted in 18b |
| Rv1572c | Conserved hypothetical protein | Insertion seqs and phages | Deleted in 18b |
| Rv1573 | Probable PhiRv1 phage protein | Insertion seqs and phages | Deleted in 18b |
| Rv1574 | Probable PhiRv1 phage related protein | Insertion seqs and phages | Deleted in 18b |
| Rv1575 | Probable PhiRv1 phage protein | Insertion seqs and phages | Deleted in 18b |
| Rv1576c | Probable PhiRv1 phage protein | Insertion seqs and phages | Deleted in 18b |
| Rv1577c | Probable PhiRv1 phage protein | Insertion seqs and phages | Deleted in 18b |
| Rv1578c | Probable PhiRv1 phage protein | Insertion seqs and phages | Deleted in 18b |
| Rv1579c | Probable PhiRv1 phage protein | Insertion seqs and phages | Deleted in 18b |
| Rv1580c | Probable PhiRv1 phage protein | Insertion seqs and phages | Deleted in 18b |
| Rv1581c | Probable PhiRv1 phage protein | Insertion seqs and phages | Deleted in 18b |
| Rv1582c | Probable PhiRv1 phage protein | Insertion seqs and phages | Deleted in 18b |
| Rv1583c | Probable PhiRv1 phage protein | Insertion seqs and phages | Deleted in 18b |
| Rv1584c | Possible PhiRv1 phage protein | Insertion seqs and phages | Deleted in 18b |
| Rv1585c | Possible phage PhiRv1 protein | Insertion seqs and phages | Deleted in 18b |
| Rv1586c | Probable PhiRv1 integrase | Insertion seqs and phages | Deleted in 18b |
| Rv1755c | Probable phospholipase C 4 (fragment) PlcD | Intermediary metabolism and respiration | Deleted in 18b |
| Rv1763 | Putative transposase for insertion sequence element IS6110 (fragment) | Insertion seqs and phages | Deleted in 18b |
| Rv1764 | Putative transposase | Insertion seqs and phages | Deleted in 18b |
| Rv1758 | Probable cutinase Cut1 | Cell wall and cell processes | Deleted in 18b |
| Rv1759c | PE-PGRS family protein Wag22 | PE/PPE | Deleted in 18b |
| Rv1760 | Possible triacylglycerol synthase (diacylglycerol acyltransferase) | Lipid metabolism | Deleted in 18b |
| Rv1761c | Possible exported protein | Cell wall and cell processes | Deleted in 18b |
| Rv1762c | Unknown protein | Conserved hypotheticals | Deleted in 18b |
| Rv1765c | Conserved hypothetical protein | Conserved hypotheticals | Deleted in 18b |
| Rv2105 | Putative transposase for insertion sequence element IS6110 (fragment) | Insertion seqs and phages | Deleted in 18b |
| Rv2106 | Probable transposase | Insertion seqs and phages | Deleted in 18b |
| Rv2167c | Probable transposase | Insertion seqs and phages | Deleted in 18b |
| Rv2168c | Putative transposase for insertion sequence element IS6110 (fragment) | Insertion seqs and phages | Deleted in 18b |
| Rv2278 | Putative transposase for insertion sequence element IS6110 (fragment) | Insertion seqs and phages | Deleted in 18b |
| Rv2279 | Probable transposase | Insertion seqs and phages | Deleted in 18b |
| Rv2479c | Probable transposase | Insertion seqs and phages | Deleted in 18b |
| Rv2480c | Possible transposase for insertion sequence element IS6110 (fragment) | Insertion seqs and phages | Deleted in 18b |
| Rv2648 | Probable transposase for insertion sequence element IS6110 (fragment) | Insertion seqs and phages | Deleted in 18b |
| Rv2649 | Probable transposase for insertion sequence element IS6110 | Insertion seqs and phages | Deleted in 18b |
| Rv2816c | Conserved hypothetical protein | Conserved hypotheticals | Deleted in 18b |
| Rv2817c | Conserved hypothetical protein | Conserved hypotheticals | Deleted in 18b |
| Rv2818c | Hypothetical protein | Unknown | Deleted in 18b |
| Rv2819c | Hypothetical protein | Conserved hypotheticals | Deleted in 18b |
| Rv3184 | Probable transposase for insertion sequence element IS6110 (fragment) | Insertion seqs and phages | Deleted in 18b |
| Rv3185 | Probable transposase | Insertion seqs and phages | Deleted in 18b |
| Rv3186 | Probable transposase for insertion sequence element IS6110 (fragment) | Insertion seqs and phages | Deleted in 18b |
| Rv3187 | Probable transposase | Insertion seqs and phages | Deleted in 18b |
| Rv3474 | Possible transposase for insertion element IS6110 (fragment) | Insertion seqs and phages | Deleted in 18b |
| Rv3475 | Possible transposase for insertion element IS6110 [second part] | Insertion seqs and phages | Deleted in 18b |
| Rv1434 | Hypothetical protein | Conserved hypotheticals | Not annotated in 18b (too short, no RNA-seq support.) |
| Rv2060 | Possible conserved integral membrane protein | Cell wall and cell processes | Nested with Rv2059. Bad prediction in H37Rv? |
| Rv3599c | Hypothetical short protein | Unknown | Not annotated in 18b, this annotation in H37Rv is suspicious. (27 aa protein?) |
| Rv3512 | PE-PGRS family protein PE_PGRS56 | PE/PPE | Assembly gap |
| Rv3513c | Probable fatty-acid-CoA ligase FadD18 (fragment) | Lipid metabolism | Assembly gap |
| Rv3514 | PE-PGRS family protein PE_PGRS57 | PE/PPE | Partially inside the assembly gap. |
