# Supplementary material for: Genomic and transcriptomic analysis of the streptomycin-dependent Mycobacterium tuberculosis strain 18b
Source: BMC Genomics. 2016 Mar 5;17:190. doi: 10.1186/s12864-016-2528-2 (PMC4779234; doi:10.1186/s12864-016-2528-2)
Supplement: Additional file 4: Table S4. — Genes from 18b that are deleted in the genome of H37Rv. (DOCX 16 kb) [file 12864_2016_2528_MOESM4_ESM.docx]

Table S4: Genes from the genome of 18b that are deleted in the genome of H37Rv.

| Gene | Length | Product | Note |
| --- | --- | --- | --- |
| MT18B_0002 | 108 | Putative transposase | IS6110_copy-1 |
| MT18B_0003 | 294 | Transposase | IS6110_copy-1 |
| MT18B_0964 | 187 | Hypothetical protein | Partially deleted in H37Rv |
| MT18B_1746 | 532 | Adenylate cyclase | 4x tandem copies in 18b, 3x in H37Rv (copies are not identical) |
| MT18B_1936 | 294 | Transposase | IS6110_copy-3 |
| MT18B_1937 | 108 | Putative transposase | IS6110_copy-3 |
| MT18B_2494 | 294 | Transposase | IS6110_copy-5 |
| MT18B_2495 | 108 | Putative transposase | IS6110_copy-5 |
| MT18B_2574 | 163 | PE family protein |  |
| MT18B_2577 | 249 | Hypothetical protein |  |
| MT18B_2578 | 248 | Hypothetical protein |  |
| MT18B_2648 | 294 | Transposase | IS6110_copy-6 |
| MT18B_2651 | 108 | Putative transposase | IS6110_copy-6 |
| MT18B_2662 | 108 | Putative transposase | IS6110_copy-7 |
| MT18B_2663 | 294 | Transposase | IS6110_copy-7 |
| MT18B_2665 | 311 | Hypothetical protein |  |
| MT18B_3720 | 108 | Putative transposase | IS6110_copy-9 |
| MT18B_3721 | 294 | Transposase | IS6110_copy-9 |
| MT18B_4006 | 294 | Transposase | IS6110_copy-11 |
| MT18B_4007 | 108 | Putative transposase | IS6110_copy-11 |
| MT18B_4156 | 294 | Transposase | IS6110_copy-12 |
| MT18B_4158 | 108 | Putative transposase | IS6110_copy-12 |
| MT18B_4204 | 633 | PPE family protein |  |
| MT18B_4415 | 378 | Molybdenum cofactor biosynthesis protein subunit MoaA |  |
| MT18B_4420 | 381 | Hypothetical protein |  |
| MT18B_4559 | 294 | Transposase | IS6110_copy-15 |
| MT18B_5304 | 108 | Putative transposase | IS6110_copy-15 |
| MT18B_4564 | 180 | PPE family protein | Duplication in 18b |
| MT18B_4786 | 387 | Hypothetical protein |  |
| MT18B_4880 | 182 | Hypothetical protein |  |
