# Supplementary material for: Genomic and transcriptomic analysis of the streptomycin-dependent Mycobacterium tuberculosis strain 18b
Source: BMC Genomics. 2016 Mar 5;17:190. doi: 10.1186/s12864-016-2528-2 (PMC4779234; doi:10.1186/s12864-016-2528-2)
Supplement: Additional file 5: Table S5. — Protein coding genes that are annotated as functional in H37Rv and as pseudogenes in 18b. (DOCX 21 kb) [file 12864_2016_2528_MOESM5_ESM.docx]

Table S5: Protein coding genes that are annotated as functional in H37Rv and as pseudogenes in 18b.

| H37Rv CDS | 18b pseudogene homolog | Product | Note |
| --- | --- | --- | --- |
| Rv0031 | MT18B_5201 | Possible remnant of a transposase | Should be a pseudogene in H37Rv? |
| Rv0045c | MT18B_0067 | Possible hydrolase | Frameshift in 18b. |
| Rv0104 | MT18B_0141 | Conserved hypothetical protein | Premature stop codon in 18b. |
| Rv0134 | MT18B_0184 | Possible epoxide hydrolase EphF (epoxide hydratase) (arene-oxide hydratase) | Frameshift in 18b. |
| Rv0606 | MT18B_0762 | Possible transposase (fragment) | Should be a pseudogene in H37Rv? |
| Rv0618 | MT18B_0782 | Probable galactose-1-phosphate uridylyltransferase GalTa [first part] | Rv0618-Rv0619 might be a single pseudogene (as in the rest of the Mtb complex). |
| Rv0619 |  |  |  |
| Rv0755A | MT18B_0980 | Putative transposase (fragment) | Should be a pseudogene in H37Rv? |
| Rv0829 | MT18B_1070 | Possible transposase (fragment) | Should be a pseudogene in H37Rv? |
| Rv0850 | MT18B_1108 | Putative transposase (fragment) | Should be a pseudogene in H37Rv? |
| Rv0905 | MT18B_1177 | Possible enoyl-CoA hydratase EchA6 (enoyl hydrase) (unsaturated acyl-CoA hydratase) (crotonase) | Frameshift in 18b. |
| Rv1034c | MT18B_1356 | Probable transposase (fragment) | Rv1034c-Rv1035c-Rv1036c might be a single pseudo-transposase. |
| Rv1035c |  |  |  |
| Rv1036c |  |  |  |
| Rv1043c | MT18B_5285 | Conserved hypothetical protein | Frameshift in 18b. |
| Rv1045 | MT18B_1376 | Hypothetical protein | Frameshift in 18b. |
| Rv1088 | MT18B_1437 | PE family protein | Rv1088-Rv1089 might be a single pseudogene. |
| Rv1089 |  |  |  |
| Rv1105 | MT18B_1462 | Possible para-nitrobenzyl esterase (fragment) | Should be a longer pseudogene in H37Rv? |
| Rv1128c | MT18B_5283 | Conserved hypothetical protein | Frameshift in 18b. |
| Rv1258c | MT18B_1667 | Probable conserved integral membrane transport protein | Frameshift in 18b. |
| Rv1280c | MT18B_1693 | Probable periplasmic oligopeptide-binding lipoprotein OppA | Frameshift in 18b. |
| Rv1370c | MT18B_1811 | Putative transposase for insertion sequence element IS6110 (fragment) | Should be a pseudogene in H37Rv? |
| Rv1511 | MT18B_1993 | GDP-D-mannose dehydratase GmdA (GDP-mannose 4,6 dehydratase) (GMD) | Frameshift in 18b. |
| Rv1549 | MT18B_5226 | Possible fatty-acid-CoA ligase FadD11.1 (fatty-acid-CoA synthetase) (fatty-acid-CoA synthase) | Rv1549-Rv1550 might be a single pseudogene. |
| Rv1550 |  |  |  |
| Rv1551 | MT18B_2045 | Possible acyltransferase PlsB1 | Frameshift in 18b. |
| Rv1770 | MT18B_2302 | Conserved protein | Frameshift in 18b. |
| Rv1917c | MT18B_2493 | PPE family protein PPE34 | Insertion of mobile element in 18b. |
|  | MT18B_2496 |  |  |
| Rv1931c | MT18B_2514 | Probable transcriptional regulatory protein | Despite an intact ORF, a conserved domain is frameshifted in both 18b and H37Rv. |
| Rv1997 | MT18B_2615 | Probable metal cation transporter P-type ATPase A CtpF | Frameshift in 18b. |
| Rv2027c | MT18B_2671 | Two component sensor histidine kinase DosT | Frameshift in 18b. |
| Rv2123 | MT18B_5305 | PPE family protein | Frameshift in 18b. |
| Rv2183c | MT18B_2878 | Conserved protein | Frameshift in 18b. |
| Rv2227 | MT18B_2927 | Conserved hypothetical protein | Despite an intact ORF, a conserved domain is frameshifted in both 18b and H37Rv (but not in strain RGTB423). |
| Rv2321c | MT18B_3065 | Probable ornithine aminotransferase RocD | Rv2321c-Rv2322c might be a single pseudogene. |
| Rv2322c |  |  |  |
| Rv2355 | MT18B_3111 | Transposase | Frameshift in 18b. |
| Rv2545 | MT18B_3384 | Possible antitoxin VapB18 | Frameshift in 18b. |
| Rv3349c | MT18B_5288 | Transposase | Should be a longer pseudogene in H37Rv? |
| Rv3383c | MT18B_4496 | Possible polyprenyl synthetase IdsB (polyprenyl transferase) (polyprenyl diphosphate synthase) | Insertion of mobile element in 18b. |
|  | MT18B_4501 |  |  |
| Rv3425 | MT18B_5271 | PPE family protein PPE57 | Frameshift in 18b. |
| Rv3507 | MT18B_4661 | PE-PGRS family protein PE_PGRS53 | Frameshift in 18b. |
| Rv3770A | MT18B_5000 | Probable remnant of a transposase | Rv3770A-Rv3770B might be a single pseudogene. |
| Rv3770B |  |  |  |
| Rv3785 | MT18B_5277 | Hypothetical protein | Frameshift in 18b. |
| Rv3894c | MT18B_5163 | ESX conserved component EccC2. ESX-2 type VII secretion system protein. Possible membrane protein. | Frameshift in 18b. |
