# Supplementary material for: Genomic and transcriptomic analysis of the streptomycin-dependent Mycobacterium tuberculosis strain 18b
Source: BMC Genomics. 2016 Mar 5;17:190. doi: 10.1186/s12864-016-2528-2 (PMC4779234; doi:10.1186/s12864-016-2528-2)
Supplement: Additional file 6: Table S6. — Genes in the genome of 18b that span two genes in H37Rv. (DOCX 24 kb) [file 12864_2016_2528_MOESM6_ESM.docx]

Table S6: Genes in the genome of 18b that span over two genes in H37Rv.

| 18b | 18b feature | H37Rv | H37Rv feature | Product |
| --- | --- | --- | --- | --- |
| MT18B_0379 | CDS | Rv0304c | CDS | PPE family protein PPE5 |
|  |  | Rv0305c | CDS | PPE family protein PPE6 |
| MT18B_0404 | CDS | Rv0325 | CDS | Hypothetical protein |
|  |  | Rv0326 | CDS | Hypothetical protein |
| MT18B_0480 | CDS | Rv0387c | CDS | Conserved hypothetical protein |
|  |  | Rv0388c | CDS | PPE family protein PPE9 |
| MT18B_0740 | CDS | Rv0590 | CDS | Mce-family protein Mce2B |
|  |  | Rv0590A | CDS | Mce-family related protein |
| MT18B_0782 | pseudogene | Rv0618 | CDS | GalTa [first part] |
|  |  | Rv0619 | CDS | GalTb [second part] |
| MT18B_1009 | CDS | Rv0781 | CDS | Probable protease II PtrBa [first part] |
|  |  | Rv0782 | CDS | Probable protease II PtrBb [second part] |
| MT18B_1356 | pseudogene | Rv1034c | CDS | Probable transposase (fragment) |
|  |  | Rv1035c | CDS | Probable transposase (fragment) |
|  |  | Rv1036c | CDS | Probable IS1560 transposase (fragment) |
| MT18B_1393 | pseudogene | Rv1054 | CDS | Probable integrase (fragment) |
|  |  | Rv1055 | CDS | Possible integrase (fragment) |
| MT18B_1437 | pseudogene | Rv1088 | CDS | PE family protein PE9 |
|  |  | Rv1089 | CDS | PE family protein PE10 |
| MT18B_1443 | CDS | Rv1089A | CDS | Probable cellulase CelA2a |
|  |  | Rv1090 | CDS | Probable cellulase CelA2b |
| MT18B_1520 | CDS | Rv1146 | CDS | Probable conserved transmembrane transport protein MmpL13b |
|  |  | Rv1145 | CDS | Probable conserved transmembrane transport protein MmpL13a |
| MT18B_1565 | CDS | Rv1180 | CDS | Probable polyketide beta-ketoacyl synthase Pks3 |
|  |  | Rv1181 | CDS | Probable polyketide beta-ketoacyl synthase Pks4 |
| MT18B_2170 | CDS | Rv1667c | CDS | Probable second part of macrolide-transport ATP-binding protein ABC transporter |
|  |  | Rv1668c | CDS | Probable first part of macrolide-transport ATP-binding protein ABC transporter |
| MT18B_2492 | CDS | Rv1915 | CDS | Probable isocitrate lyase AceAa [first part] |
|  |  | Rv1916 | CDS | Probable isocitrate lyase AceAb [second part] |
| MT18B_2763 | CDS | Rv2098c | CDS | PE-PGRS family protein PE_PGRS36 |
|  |  | Rv2099c | CDS | PE family protein PE21 |
| MT18B_2847 | CDS | Rv2160A | CDS | Conserved hypothetical protein |
|  |  | Rv2160c | CDS | Conserved hypothetical protein |
| MT18B_2961 | CDS | Rv2250A | CDS | Possible flavoprotein |
|  |  | Rv2251 | CDS | Possible flavoprotein |
| MT18B_2972 | CDS | Rv2261c | CDS | Conserved hypothetical protein |
|  |  | Rv2262c | CDS | Conserved hypothetical protein |
| MT18B_3015 | CDS | Rv2292c | CDS | Hypothetical protein |
|  |  | Rv2293c | CDS | Conserved hypothetical protein |
| MT18B_3065 | pseudogene | Rv2321c | CDS | Probable ornithine aminotransferase (C-terminus part) RocD2 |
|  |  | Rv2322c | CDS | Probable ornithine aminotransferase (N-terminus part) RocD1 |
| MT18B_3361 | CDS | Rv2526 | CDS | Possible antitoxin VapB17 |
|  |  | Rv2527 | CDS | Possible toxin VapC17 |
| MT18B_3406 | CDS | Rv2561 | CDS | Conserved hypothetical protein |
|  |  | Rv2562 | CDS | Conserved hypothetical protein |
| MT18B_3810 | CDS | Rv2879c | CDS | Conserved hypothetical protein |
|  |  | Rv2880c | CDS | Conserved hypothetical protein |
| MT18B_3901 | CDS | Rv2946c | CDS | Probable polyketide synthase Pks1 |
|  |  | Rv2947c | CDS | Probable polyketide synthase Pks15 |
| MT18B_3943 | CDS | Rv2974c | CDS | Conserved hypothetical alanine rich protein |
|  |  | Rv2975c | CDS | Conserved hypothetical protein |
| MT18B_4012 | CDS | Rv3021c | pseudogene | PPE family protein PPE47 |
|  |  | Rv3022c | pseudogene | PPE family protein PPE48 |
| MT18B_4308 | CDS | Rv3233c | CDS | Possible triacylglycerol synthase (diacylglycerol acyltransferase) |
|  |  | Rv3234c | CDS | Putative triacylglycerol synthase (diacylglycerol acyltransferase) Tgs3 |
| MT18B_4447 | CDS | Rv3344c | CDS | PE-PGRS family protein PE_PGRS49 |
|  |  | Rv3345c | CDS | PE-PGRS family protein PE_PGRS50 |
| MT18B_4597 | CDS | Rv3453 | CDS | Possible conserved transmembrane protein |
|  |  | Rv3454 | CDS | Probable conserved integral membrane protein |
| MT18B_4729 | CDS | Rv3566c | CDS | Arylamine N-acetyltransferase Nat |
|  |  | Rv3566A | CDS | Hypothetical protein |
| MT18B_4841 | CDS | Rv3652 | CDS | PE-PGRS family-related protein PE_PGRS60 |
|  |  | Rv3653 | CDS | PE-PGRS family-related protein PE_PGRS61 |
| MT18B_5000 | pseudogene | Rv3770A | CDS | Probable remnant of a transposase |
|  | pseudogene | Rv3770B | CDS | Probable remnant of a transposase |
| MT18B_5077 | CDS | Rv3829c | CDS | Probable dehydrogenase |
|  |  | Rv3830c | CDS | Transcriptional regulatory protein (probably TetR-family) |
| MT18B_5168 | CDS | Rv3897c | CDS | Conserved hypothetical protein |
|  |  | Rv3898c | CDS | Conserved hypothetical protein |
| MT18B_5226 | pseudogene | Rv1549 | CDS | Possible fatty-acid-CoA ligase FadD11.1 |
|  |  | Rv1550 | CDS | Probable fatty-acid-CoA ligase FadD11 |
| MT18B_5302 | pseudogene | Rv3636 | CDS | Possible transposase |
|  |  | Rv3637 | CDS | Possible transposase |
