# Supplementary material for: Genomic and transcriptomic analysis of the streptomycin-dependent Mycobacterium tuberculosis strain 18b
Source: BMC Genomics. 2016 Mar 5;17:190. doi: 10.1186/s12864-016-2528-2 (PMC4779234; doi:10.1186/s12864-016-2528-2)
Supplement: Additional file 7: Table S7. — Positions of IS6110 in the genome sequences of M. tuberculosis 18b and H37Rv. (DOCX 27 kb) [file 12864_2016_2528_MOESM7_ESM.docx]

Table S7: Positions of IS6110 transposons in the reference genome sequences of *Mycobacterium tuberculosis* 18b and H37Rv.

| **18b** | | | | | **H37Rv** | | | | |  |
| --- | --- | --- | --- | --- | --- | --- | --- | --- | --- | --- |
| IS name | Coordinates | Str. | Size | Gene | IS name | Coordinates | Str. | Size | Gene | Note |
| IS6110_copy-1 | 1595-2949 | + | 1355 | MT18B_0002 |  |  |  |  |  | Deleted in H37Rv |
|  |  |  |  | MT18B_0003 |  |  |  |  |  |  |
| IS6110_copy-2 | 1541931-1542567 | - | 637 | MT18B_1811* | IS6110-2 | 1541952-1543306 | - | 1355 | Rv1370c | Truncated in 18b |
|  |  |  |  |  |  |  |  |  | Rv1369c |  |
| IS6110_copy-3 | 1656061-1657415 | - | 1355 | MT18B_1936 |  |  |  |  |  | Deleted in H37Rv |
|  |  |  |  | MT18B_1937 |  |  |  |  |  |  |
| IS6110_copy-4 | 1978472-1979826 | - | 1355 | MT18B_2285 | IS6110-3 | 1987703-1989057 | - | 1355 | Rv1756c | *plcD*-*Rv1762c* region deleted in 18b, only the IS remains. |
|  |  |  |  | MT18B_2286 |  |  |  |  | Rv1757c |  |
| IS6110_copy-5 | 2147668-2149022 | - | 1355 | MT18B_2494 |  |  |  |  |  | Deleted in H37Rv |
|  |  |  |  | MT18B_2495 |  |  |  |  |  |  |
| IS6110_copy-6 | 2251442-2252796 | - | 1355 | MT18B_2648 |  |  |  |  |  | Deleted in H37Rv |
|  |  |  |  | MT18B_2651 |  |  |  |  |  |  |
| IS6110_copy-7 | 2258271-2259625 | + | 1355 | MT18B_2662 |  |  |  |  |  | Deleted in H37Rv |
|  |  |  |  | MT18B_2663 |  |  |  |  |  |  |
| IS6110_copy-8 | 2625877-2627229 | - | 1353 | MT18B_3112 | IS6110-8 | 2635577-2636931 | + | 1355 | Rv2354 | Inverted in 18b |
|  |  |  |  | MT18B_3111 |  |  |  |  | Rv2355 |  |
| IS6110_copy-9 | 3105240-3106594 | + | 1355 | MT18B_3720 |  |  |  |  |  | Deleted in H37Rv |
|  |  |  |  | MT18B_3721 |  |  |  |  |  |  |
| IS6110_copy-10 | 3111848-3113202 | + | 1355 | MT18B_3730 | IS6110-11 | 3120523-3121897 | - | 1375 | Rv2814c | Inverted in 18b |
|  |  |  |  | MT18B_3729 |  |  |  |  | Rv2815c |  |
| IS6110_copy-11 | 3364672-3366026 | - | 1355 | MT18B_4006 |  |  |  |  |  | Deleted in H37Rv |
|  |  |  |  | MT18B_4007 |  |  |  |  |  |  |
| IS6110_copy-12 | 3481745-3483099 | - | 1355 | MT18B_4156 |  |  |  |  |  | Deleted in H37Rv |
|  |  |  |  | MT18B_4158 |  |  |  |  |  |  |
| IS6110_copy-13 | 3703446-3704800 | - | 1355 | MT18B_4423 | IS6110-14 | 3710382-3711736 | + | 1355 | Rv3325 | Inverted in 18b |
|  |  |  |  | MT18B_4422 |  |  |  |  | Rv3326 |  |
| IS6110_copy-14 | 3793427-3794781 | - | 1355 | MT18B_4498 | IS6110-15 | 3795058-3796412 | + | 1355 | Rv3380c | Inverted in 18b and slightly translocated |
|  |  |  |  | MT18B_4497 |  |  |  |  | Rv3381c |  |
| IS6110_copy-15 | 3842028-3843382 | - | 1355 | MT18B_4559 |  |  |  |  |  | Deleted in H37Rv |
|  |  |  |  | MT18B_5304 |  |  |  |  |  |  |
|  |  |  |  |  | IS6110-1 | 889021-890375 | + | 1355 | Rv0795 | Deleted in 18b |
|  |  |  |  |  |  |  |  |  | Rv0796 |  |
|  |  |  |  |  | IS6110-4 | 1996101-1997455 | + | 1355 | Rv1763 | Deleted in 18b |
|  |  |  |  |  |  |  |  |  | Rv1764 |  |
|  |  |  |  |  | IS6110-5 | 2365414-2366768 | + | 1355 | Rv2105 | Deleted in 18b |
|  |  |  |  |  |  |  |  |  | Rv2106 |  |
|  |  |  |  |  | IS6110-6 | 2430117-2431471 | - | 1355 | Rv2167c | Deleted in 18b |
|  |  |  |  |  |  |  |  |  | Rv2168c |  |
|  |  |  |  |  | IS6110-7 | 2550014-2551368 | + | 1355 | Rv2278 | Deleted in 18b |
|  |  |  |  |  |  |  |  |  | Rv2279 |  |
|  |  |  |  |  | IS6110-9 | 2784614-2785970 | - | 1357 | Rv2479c | Deleted in 18b |
|  |  |  |  |  |  |  |  |  | Rv2480c |  |
|  |  |  |  |  | IS6110-10 | 2972109-2973463 | + | 1355 | Rv2648 | Deleted in 18b |
|  |  |  |  |  |  |  |  |  | Rv2649 |  |
|  |  |  |  |  | IS6110-12 | 3551230-3552584 | + | 1355 | Rv3184 | Deleted in 18b |
|  |  |  |  |  |  |  |  |  | Rv3185 |  |
|  |  |  |  |  | IS6110-13 | 3552713-3554067 | + | 1355 | Rv3186 | Deleted in 18b |
|  |  |  |  |  |  |  |  |  | Rv3187 |  |
|  |  |  |  |  | IS6110-16 | 3890779-3892133 | + | 1355 | Rv3474 | Deleted in 18b |
|  |  |  |  |  |  |  |  |  | Rv3475 |  |

*Pseudogene.
