# Supplementary material for: Genomic and transcriptomic analysis of the streptomycin-dependent Mycobacterium tuberculosis strain 18b
Source: BMC Genomics. 2016 Mar 5;17:190. doi: 10.1186/s12864-016-2528-2 (PMC4779234; doi:10.1186/s12864-016-2528-2)
Supplement: Additional file 8: Table S8. — SNPs unique to M. tuberculosis 18b from a comparison with 1793 M. tuberculosis genomes. (DOCX 25 kb) [file 12864_2016_2528_MOESM8_ESM.docx]

Table S8: SNPs unique to the genome of *Mycobacterium tuberculosis* 18b, based on a comparison with 1,793 *M. tuberculosis* genomes.

| Position in H37Rv | Position in 18b | H37Rv | 18b | SNP type | Protein change | Gene | Product |
| --- | --- | --- | --- | --- | --- | --- | --- |
| 34,891 | 36,230 | G | A | Synonymous | Pro199Pro | *bioF2* | Possible 8-amino-7-oxononanoate synthase BioF2 |
| 84,507 | 82,753 | A | G | Missense | Asn171Ser | *Rv0075* | Probable aminotransferase |
| 115,092 | 113,333 | C | G | Missense | Leu1698Val | *nrp* | Probable peptide synthetase Nrp |
| 262,170 | 260,579 | A | G | Missense | Tyr416Cys | *Rv0218* | Probable conserved transmembrane protein |
| 288,294 | 286,632 | A | G | Missense | Asp370Gly | *lpqI* | Probable conserved lipoprotein LpqI |
| 440,128 | 438,523 | C | T | Synonymous | Ala86Ala | *mgtE* | Possible Mg2+ transport transmembrane protein MgtE |
| 459,579 | 457,974 | G | A | Synonymous | Arg808Arg | *clpB* | Probable endopeptidase ATP binding protein (chain B) ClpB (heat shock protein F84.1) |
| 489,603 | 487,999 | C | T | Synonymous | Gly1291Gly | *pks6* | Probable membrane bound polyketide synthase Pks6 |
| 609,957 | 608,319 | C | T | Synonymous | Val404Val | *Rv0517* | Possible membrane acyltransferase |
| 620,853 | 619,215 | G | A | Synonymous | Ala321Ala | *ccsA* | Possible cytochrome C-type biogenesis protein CcsA |
| 724,557 | 723,004 | T | G | Missense | Thr153Pro | *recB* | Probable exonuclease V (beta chain) RecB |
| 789,037 | 788,017 | C | G | Stop gained | Tyr366* | *Rv0688* | Putative ferredoxin reductase |
| 876,078 | 875,265 | G | C | Synonymous | Val449Val | *ptrBb* | Probable protease II PtrBb [second part] |
| 878,989 | 878,176 | G | T | Missense | Gly118Cys | *Rv0784* | Conserved hypothetical protein |
| 927,696 | 925,522 | C | G | Intergenic | - | *-* | - |
| 1,050,470 | 1,048,303 | C | A | Intergenic | - | *-* | - |
| 1,079,193 | 1,077,026 | G | A | Missense | Ala151Thr | *ctpV* | Probable metal cation transporter P-type ATPase CtpV |
| 1,167,232 | 1,165,064 | A | G | Synonymous | Arg60Arg | *Rv1044* | Conserved hypothetical protein |
| 1,200,316 | 1,198,304 | C | T | Missense | Gly19Ser | *Rv1075c* | Conserved exported protein |
| 1,206,121 | 1,204,109 | T | G | Synonymous | Arg100Arg | *Rv1081c* | Probable conserved membrane protein |
| 1,266,601 | 1,264,584 | A | G | Missense | Phe129Leu | *Rv1139c* | Conserved hypothetical membrane protein |
| 1,337,795 | 1,335,780 | G | T | Missense | Ala240Glu | *Rv1194c* | Conserved protein |
| 1,569,105 | 1,567,704 | G | A | Synonymous | Arg161Arg | *Rv1393c* | Probable monoxygenase |
| 1,592,894 | 1,591,493 | C | T | Missense | Pro86Ser | *lprH* | Probable lipoprotein LprH |
| 1,651,435 | 1,650,480 | C | T | Synonymous | Asp240Asp | *Rv1463* | Probable conserved ATP-binding protein ABC transporter |
| 1,661,654 | 1,662,057 | C | T | Synonymous | Arg333Arg | *Rv1473* | Probable macrolide-transport ATP-binding protein ABC transporter |
| 1,668,167 | 1,668,570 | G | T | Missense | Gly393Val | *ripA* | Peptidoglycan hydrolase |
| 1,805,989 | 1,797,143 | C | T | Missense | Pro113Ser | *hisI* | Probable phosphoribosyl-AMP 1,6 cyclohydrolase HisI |
| 1,805,990 | 1,797,144 | C | T | Missense | Pro113Leu | *hisI* | Probable phosphoribosyl-AMP 1,6 cyclohydrolase HisI |
| 1,911,285 | 1,902,470 | T | G | Synonymous | Arg39Arg | *Rv1685c* | Conserved hypothetical protein |
| 1,969,495 | 1,960,791 | C | T | Synonymous | Thr164Thr | *pknE* | Probable transmembrane serine/threonine-protein kinase E PknE |
| 2,075,230 | 2,056,329 | C | T | Synonymous | Ile130Ile | *Rv1830* | Conserved hypothetical protein |
| 2,181,968 | 2,165,287 | A | C | Missense | Ser159Ala | *Rv1930c* | Conserved hypothetical protein |
| 2,193,506 | 2,176,825 | G | A | Missense | Ala301Thr | *ribA1* | Probable riboflavin biosynthesis protein RibA1 |
| 2,217,302 | 2,200,622 | G | A | Missense | Ala47Thr | *Rv1973* | Possible conserved Mce associated membrane protein |
| 2,316,709 | 2,312,238 | A | C | Stop lost | *512Cys | *Rv2059* | Conserved hypothetical protein |
| 2,329,631 | 2,325,160 | G | A | Synonymous | Gly172Gly | *cobL* | Precorrin-6Y C(5,15)-methyltransferase (decarboxylating) CobL |
| 2,373,644 | 2,367,949 | G | C | Missense | Gly339Arg | *Rv2113* | Probable integral membrane protein |
| 2,373,646 | 2,367,951 | C | G | Synonymous | Gly339Gly | *Rv2113* | Probable integral membrane protein |
| 2,428,202 | 2,422,626 | T | C | Missense | Ser13Gly | *Rv2164c* | Probable conserved proline rich membrane protein |
| 2,435,440 | 2,428,506 | C | T | Synonymous | Tyr198Tyr | *idsA2* | Probable geranylgeranyl pyrophosphate synthetase IdsA2 |
| 2,623,078 | 2,614,905 | G | A | Synonymous | Asp225Asp | *dgt* | Probable deoxyguanosine triphosphate triphosphohydrolase Dgt |
| 2,629,827 | 2,621,654 | G | C | Missense | His165Asp | *plcB* | Membrane-associated phospholipase C 2 PlcB |
| 2,730,675 | 2,723,325 | A | G | Missense | Ile692Thr | *Rv2435c* | Probable cyclase (adenylyl- or guanylyl-)(adenylate- or guanylate-) |
| 2,787,596 | 2,778,887 | A | G | Missense | Val563Ala | *plsB2* | Probable glycerol-3-phosphate acyltransferase PlsB2 |
| 2,825,712 | 2,817,003 | G | C | Missense | Gln460Glu | *Rv2510c* | Conserved protein |
| 2,937,653 | 2,928,940 | C | T | Synonymous | Leu71Leu | *Rv2609c* | Probable conserved membrane protein |
| 2,940,188 | 2,931,475 | C | A | Missense | Gly142Val | *pgsA1* | PI synthase PgsA1 |
| 2,984,660 | 2,974,587 | G | A | Intergenic | - | *-* | - |
| 3,178,555 | 3,163,835 | C | A | Missense | Ser253Ile | *Rv2867c* | GCN5-related N-acetyltransferase |
| 3,186,008 | 3,171,288 | A | C | Missense | Ser388Arg | *dipZ* | Possible integral membrane C-type cytochrome biogenesis protein DipZ |
| 3,265,842 | 3,251,840 | C | T | Synonymous | Leu1199Leu | *ppsD* | Phenolpthiocerol synthesis type-I polyketide synthase PpsD |
| 3,294,116 | 3,280,114 | G | A | Synonymous | Gly746Gly | *pks1* | Probable polyketide synthase Pks1 |
| 3,318,355 | 3,304,360 | T | C | Missense | Asp154Gly | *kdtB* | Probable phosphopantetheine adenylyltransferase KdtB |
| 3,324,037 | 3,310,042 | T | C | Missense | Glu147Gly | *Rv2969c* | Possible conserved membrane or secreted protein |
| 3,505,332 | 3,495,559 | C | T | Intergenic | - | *-* | - |
| 3,622,843 | 3,612,501 | C | T | Synonymous | Gln83Gln | *Rv3243c* | Unknown protein |
| 3,791,699 | 3,788,654 | C | T | Missense | Ala219Thr | *Rv3377c* | Halimadienyl diphosphate synthase |
| 3,898,840 | 3,898,663 | A | C | Synonymous | Ala1007Ala | *Rv3479* | Possible transmembrane protein |
| 4,017,757 | 4,017,111 | C | T | Missense | Asp138Asn | *Rv3575c* | Transcriptional regulatory protein (probably LacI-family) |
| 4,063,052 | 4,062,961 | G | A | Missense | Asp176Asn | *lpqG* | Probable conserved lipoprotein LpqG |
| 4,139,247 | 4,139,552 | T | C | Missense | Asp170Gly | *glpK* | Probable glycerol kinase GlpK |
| 4,167,454 | 4,167,874 | G | A | Synonymous | Thr225Thr | *Rv3722c* | Conserved protein |
| 4,205,330 | 4,205,751 | G | T | Missense | Ala15Asp | *proX* | Possible osmoprotectant ProX |
| 4,332,357 | 4,332,687 | T | C | Missense | His1242Arg | *gltB* | Probable ferredoxin-dependent glutamate synthase [NADPH] (large subunit) GltB |
