# Supplementary material for: Genomic and transcriptomic analysis of the streptomycin-dependent Mycobacterium tuberculosis strain 18b
Source: BMC Genomics. 2016 Mar 5;17:190. doi: 10.1186/s12864-016-2528-2 (PMC4779234; doi:10.1186/s12864-016-2528-2)
Supplement: Additional file 11: Figure S1. — Weighted Venn diagrams representing overlap of the differential gene expression results from this work with those from stationary phase induction and response to macrophage infection. (PDF 163 kb) [file 12864_2016_2528_MOESM11_ESM.pdf]

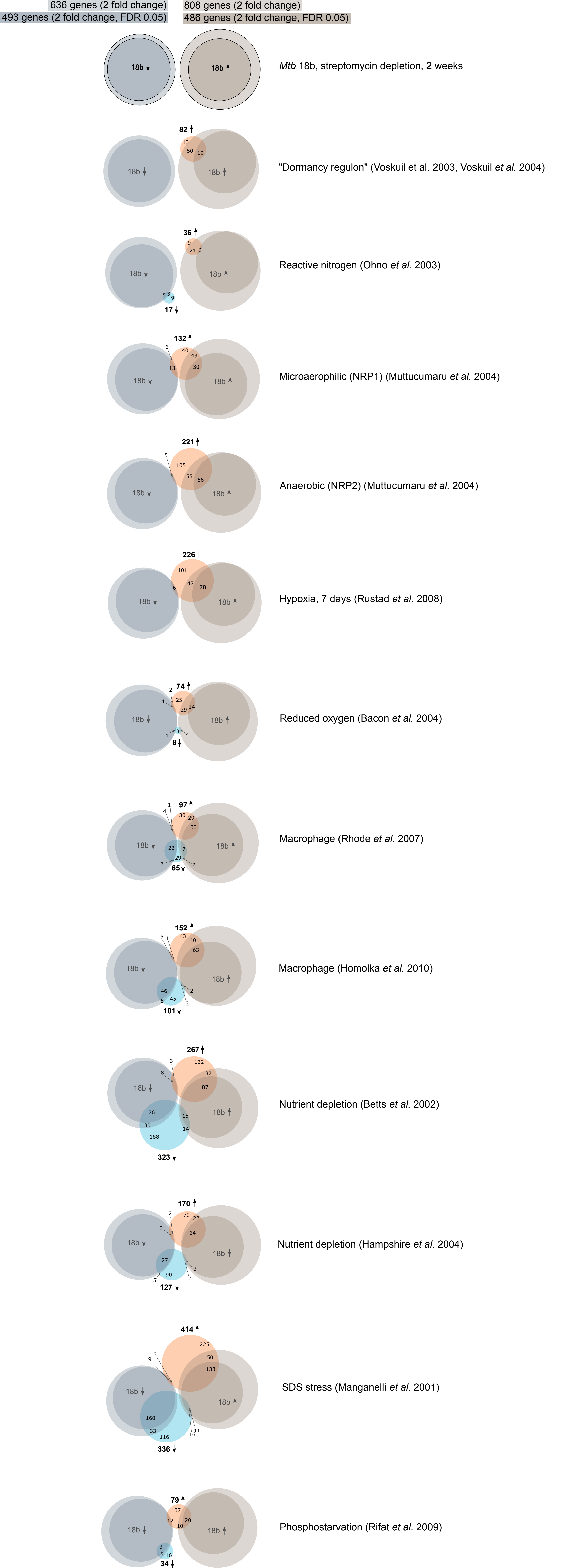
636 genes (2 fold change)
493 genes (2 fold change, FDR 0.05)
808 genes (2 fold change)
486 genes (2 fold change, FDR 0.05)
18b ↓
18b ↑
Mtb 18b, streptomycin depletion, 2 weeks
82 ↑
13
50
19
"Dormancy regulon" (Voskuil et al. 2003, Voskuil et al. 2004)
36 ↑
9
21
6
5
3
9
17 ↓
Reactive nitrogen (Ohno et al. 2003)
132 ↑
6
40
43
13
30
Microaerophilic (NRP1) (Muttucumaru et al. 2004)
221 ↑
5
105
55
56
Anaerobic (NRP2) (Muttucumaru et al. 2004)
226
101
47
6
78
Hypoxia, 7 days (Rustad et al. 2008)
74 ↑
2
25
4
29
14
3
1
4
8 ↓
Reduced oxygen (Bacon et al. 2004)
97 ↑
1
4
30
29
33
22
7
29
2
5
65 ↓
Macrophage (Rhode et al. 2007)
152 ↑
5
1
43
40
63
46
2
5
45
3
101 ↓
Macrophage (Homolka et al. 2010)
267 ↑
3
132
8
37
87
76
15
30
14
188
323 ↓
Nutrient depletion (Betts et al. 2002)
170 ↑
2
79
22
3
64
27
3
90
2
5
127 ↓
Nutrient depletion (Hampshire et al. 2004)
414 ↑
225
9
3
50
133
160
33
116
16
11
336 ↓
SDS stress (Manganelli et al. 2001)
79 ↑
37
12
20
10
3
15
16
34 ↓
Phosphostarvation (Rifat et al. 2009)
