# Supplementary figures and images for: Genomic and transcriptomic analysis of the streptomycin-dependent Mycobacterium tuberculosis strain 18b
Source: BMC Genomics. 2016 Mar 5;17:190. doi: 10.1186/s12864-016-2528-2 (PMC4779234; doi:10.1186/s12864-016-2528-2)

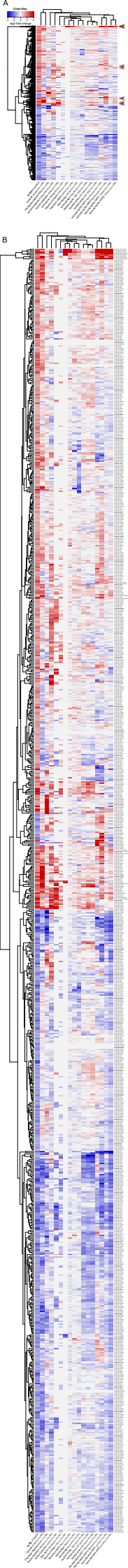

Supplement: Additional file 12: Figure S2. — Hierarchical clustering of differentially expressed genes in SS18b and from previous works showing >2-fold differences. (PDF 332 kb) [file 12864_2016_2528_MOESM12_ESM.pdf]

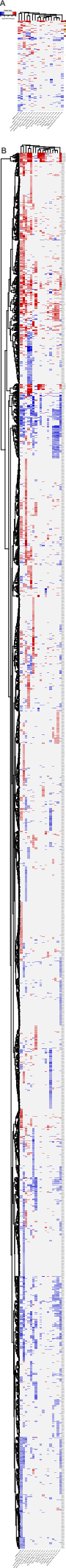

Supplement: Additional file 13: Figure S3. — Hierarchical clustering of differentially expressed genes in SS18b and the results from previous works with 2-fold differences displayed as shrunk and expanded images. (PDF 748 kb) [file 12864_2016_2528_MOESM13_ESM.pdf]

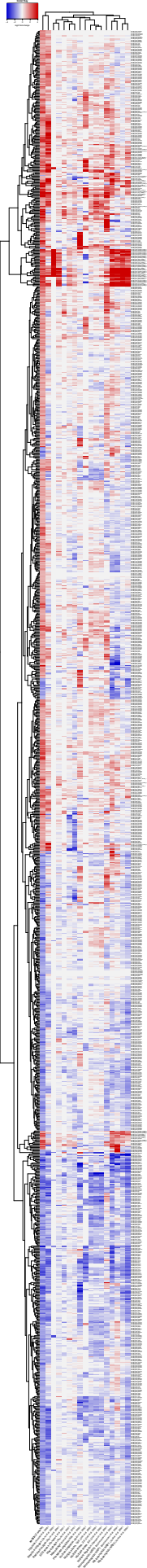

Supplement: Additional file 14: Figure S4. — Hierarchical clustering of differentially expressed genes in SS18b and previous works. Expanded version of Fig. 4 including gene names. (PDF 239 kb) [file 12864_2016_2528_MOESM14_ESM.pdf]
